# The REEP5/TRAM1 complex binds SARS-CoV-2 NSP3 and promotes virus replication

**DOI:** 10.1128/jvi.00507-23

**Published:** 2023-09-28

**Authors:** Jie Li, Qi Gui, Feng-Xia Liang, Joseph Sall, Qingyue Zhang, Yatong Duan, Avantika Dhabaria, Manor Askenazi, Beatrix Ueberheide, Kenneth A. Stapleford, Michele Pagano

**Affiliations:** 1 Department of Biochemistry and Molecular Pharmacology, New York University Grossman School of Medicine, New York, New York, USA; 2 Laura and Isaac Perlmutter NYU Cancer Center, New York University Grossman School of Medicine, New York, New York, USA; 3 Microscopy Laboratory, Division of Advanced Research Technologies, New York University Grossman School of Medicine, New York, New York, USA; 4 William A. Shine Great Neck South High School, Lake Success, New York, USA; 5 Proteomics Laboratory, Division of Advanced Research Technologies, New York University Grossman School of Medicine, New York, New York, USA; 6 Biomedical Hosting LLC, Arlington, Massachusetts, USA; 7 Department of Neurology, New York University Grossman School of Medicine, New York, New York, USA; 8 Department of Microbiology, New York University Grossman School of Medicine, New York, New York, USA; 9 Howard Hughes Medical Institute, New York University Grossman School of Medicine, New York, New York, USA; Loyola University, Chicago, Maywood, Illinois, USA

**Keywords:** coronaviruses, SARS-CoV-2, replication organelle, ER membrane rearrangements, NSP3, REEP5, TRAM1

## Abstract

Severe acute respiratory syndrome coronavirus 2 (SARS-CoV-2), like other coronaviruses, replicates their genome in virus-induced cytosolic membrane-bound replication organelles (ROs). SARS-CoV-2 promotes the biogenesis of ROs by inducing the rearrangement of endoplasmic reticulum (ER) membranes. NSP3, NSP4, and NSP6 are transmembrane viral non-structural proteins (NSPs) and essential players in the formation of ROs. To understand how these three NSPs work synergistically with host-binding proteins, we performed affinity purifications followed by mass spectrometry analyses to study the host-viral protein-protein interactome of NSP3, NSP4, and NSP6 expressed individually and in combination. Through this analysis, we identified two host transmembrane proteins, REEP5 and TRAM1, as critical interacting partners of NSP3 that localize at the membrane of the RO. REEP5 interacts with TRAM1 endogenously and binds NSP3 during SARS-CoV-2 infection. REEP5 knockout reduces ER membrane rearrangements and inhibits SARS-CoV-2 replication. Collectively, our study shows that the host REEP5/TRAM1 complex binds NSP3, promoting RO biogenesis and viral replication.

**IMPORTANCE:**

Generation of virus-host protein–protein interactions (PPIs) maps may provide clues to uncover SARS-CoV-2-hijacked cellular processes. However, these PPIs maps were created by expressing each viral protein singularly, which does not reflect the life situation in which certain viral proteins synergistically interact with host proteins. Our results reveal the host-viral protein-protein interactome of SARS-CoV-2 NSP3, NSP4, and NSP6 expressed individually or in combination. Furthermore, REEP5/TRAM1 complex interacts with NSP3 at ROs and promotes viral replication. The significance of our research is identifying virus-host interactions that may be targeted for therapeutic intervention.

## INTRODUCTION

Coronaviruses (CoVs) replicate their genomes in the cytoplasm of host cells (1, 2). This process is supported by virus-induced rearrangement of host endoplasmic reticulum (ER) membranes that generate what is known as the replication organelle (RO) (3, 4). The most abundant components of ROs for CoVs are double-membrane vesicles (DMVs), which are central hubs for viral RNA synthesis (4 – 6). Viral replicase complexes are found in DMVs and are required for replication of viral genome and translation of structural proteins (6). The concerted actions of viral-host protein–protein interactions (PPIs) are crucial for the generation of these replication platforms by hijacking various host cellular pathways involved in membrane-shaping and transportation.

CoVs have a large genome that encodes 4 structural proteins and 16 non-structural proteins (NSPs) that, together, ensure virus replication in host cells (6). The four structural proteins are spike (S), nucleocapsid (N), membrane (M), and envelope (E) proteins. The 16 NSPs include 13 cytosolic proteins and 3 transmembrane proteins, NSP3, NSP4, and NSP6. NSP3, NSP4, and NSP6 bind each other at the surface of DMVs and are crucial for the generation of the ROs (7 – 9). Co-expression of NSP3 and NSP4 is required and sufficient to induce the formation of these DMVs in human cells (8, 10), while NSP6 contributes to the established connection between ER membranes and DMVs (11).

Accumulated evidence supports the idea that viral RNA synthesis occurs inside DMVs (4, 5, 12), as it provides a dual advantage for the virus by (i) spatio-temporally optimizing the organization of cellular and viral constituents required for RNA synthesis and (ii) preventing attacks from the host anti-viral defense system. A recent study (12) visualized a molecular pore complex (~1.8 MDa intermembrane platform) that spans both membranes of the DMVs, suggesting that newly synthesized viral RNAs can travel from the lumen of DMVs to the cytosol. Moreover, the coronavirus transmembrane protein NSP3 has been validated as a component of the pore complex (12). Another study revealed that SARS-CoV-2 NSP3 and NSP4 are minimal components forming a DMV spanning pore and showed that NSP3 Ubl1-Ubl2 domains are critical for inducing membrane curvature and DMV formation (13). As proteins interacting with NSP3 (including NSP4 and NSP6) are likely part of the pore complex, identifying the host interactome of NSP3/NSP4/NSP6 proteins at DMVs is expected to shed light on the regulatory mechanism of viral RNA synthesis.

Virus-host PPIs are the vital engine of the viral life cycle after virus entry in host cells (14). Several recent studies have explored SARS-CoV-2–host PPIs by affinity purification-mass spectrometry (MS) and proximity-based labeling MS method (15 – 18). Generation of virus-host PPIs maps may provide clues to uncover SARS-CoV-2-hijacked cellular processes. However, these PPIs maps were created by expressing each viral protein singularly, which does not reflect the life situation in which certain viral proteins synergistically interact with host proteins.

In this study, we performed affinity purification followed by mass spectrometry analysis (AP-MS) to study the host-viral protein-protein interactome of SARS-CoV-2 NSP3, NSP4, and NSP6 expressed individually or in combination. We identified the REEP5/TRAM1 complex as host proteins binding NSP3 at the ROs. This study reveals a previously unknown function of the REEP5/TRAM1 complex to regulate SARS-CoV-2 RO biogenesis and replication.

## RESULTS

### Host interactome of NSP3, NSP4, and NSP6

First, we expressed SARS-CoV-2 NSP3, NSP4, and NSP6 proteins in mammalian cells. The open reading frames (Orfs) of NSP3, NSP4, and NSP6 were codon optimized with two different tools: one from Fritz Roth (FR) (19) and the other from online Rare Codon Analyzer (RCA; https://www.biologicscorp.com/tools/RareCodonAnalyzer/#.Y_6vR-zMJTZ). The optimized Orfs were fused to a 2xStrep affinity tag and cloned into a mammalian expression vector. To verify viral protein expression, we performed Western Blots with an anti-Strep antibody on whole cell extracts (WCE) from HEK293T cells transfected with the SARS-CoV-2 Orfs (Fig. S1A). The Orfs from FR displayed higher expression than the Orfs from RCA, so we used the Orfs from FR for further studies. We observed that the expression level of full-length SARS-CoV-2 NSP3 is much lower compared to NSP4, possibly due to the complex protein topology of the full-length NSP3, while the expression level of the C-terminal one-third of NSP3 (NSP3C) is comparable to NSP4 (Fig. S1A; Fig. 5D).

Next, we aimed at confirming the functionality of NSP3, NSP4, and NSP6 in mammalian cells. Since SARS-CoV and mouse hepatitis virus (MHV) share many similarities in virology and epidemiology with SARS-CoV-2, mechanistic insights learnt from SARS-CoV and MHV could offer clues into SARS-CoV-2 biology (20, 21). For both SARS-CoV and MHV, co-expression of NSP3C with NSP4 is enough to induce ER membrane rearrangements in mammalian cells (22, 23). To verify this observation with SARS-CoV-2, we co-expressed the corresponding EGFP-tagged NSP3C and mCherry-tagged NSP4 from SARS-CoV-2 together with an mTagBFP2-tagged ER marker in U-2 OS cells, which have relatively large cytoplasmic volume. Using correlative light and electron microscopy (CLEM), we confirmed the co-localization of NSP3C and NSP4 with the ER marker. Fluorescent images showed that NSP3C and NSP4 fully colocalized in ring-shaped structures, which at the EM level are corresponded to multi-membrane vesicles (MMVs) with an average size of 1–3 μm (Fig. S1B), possibly generated from the fusion of small-sized DMVs with a diameter of 150–350 nm as suggested previously (3, 4). To confirm that NSP3C binds NSP4 and NSP6, we performed affinity purification with biotin magnetic beads (AP-Strep) followed by WB in HEK293T cells co-transfected with NSP3C-2xStrep and Flag-tagged NSP4 (or its mutants), NSP6, other NSPs (NSP2 and NSP14), or host transmembrane proteins (V0A1 and V0D). We observed a specific binding of NSP3C to NSP4 and NSP6, but not to NSP2, NSP14, V0A1, and V0D (Fig. S1C). In agreement with a previous study (23), two-amino acid substitutions in NSP4 (NSP4-H120N/F121L), but not the deletion of amino acids 220–234 (NSP4-Δ220–234), reduced its binding to NSP3C compared to NSP4 wild type. Although HEK293T cells are isolated from kidney tissue, which is not a primary tissue target of SARS-CoV-2, previous work has identified host-binding factors of CoV proteins using HEK293T cells (15 – 18). Based on all of the above, we decided to express the NSP proteins in HEK293T cells to identify their host-binding proteins by AP-MS.

**Fig 1:**
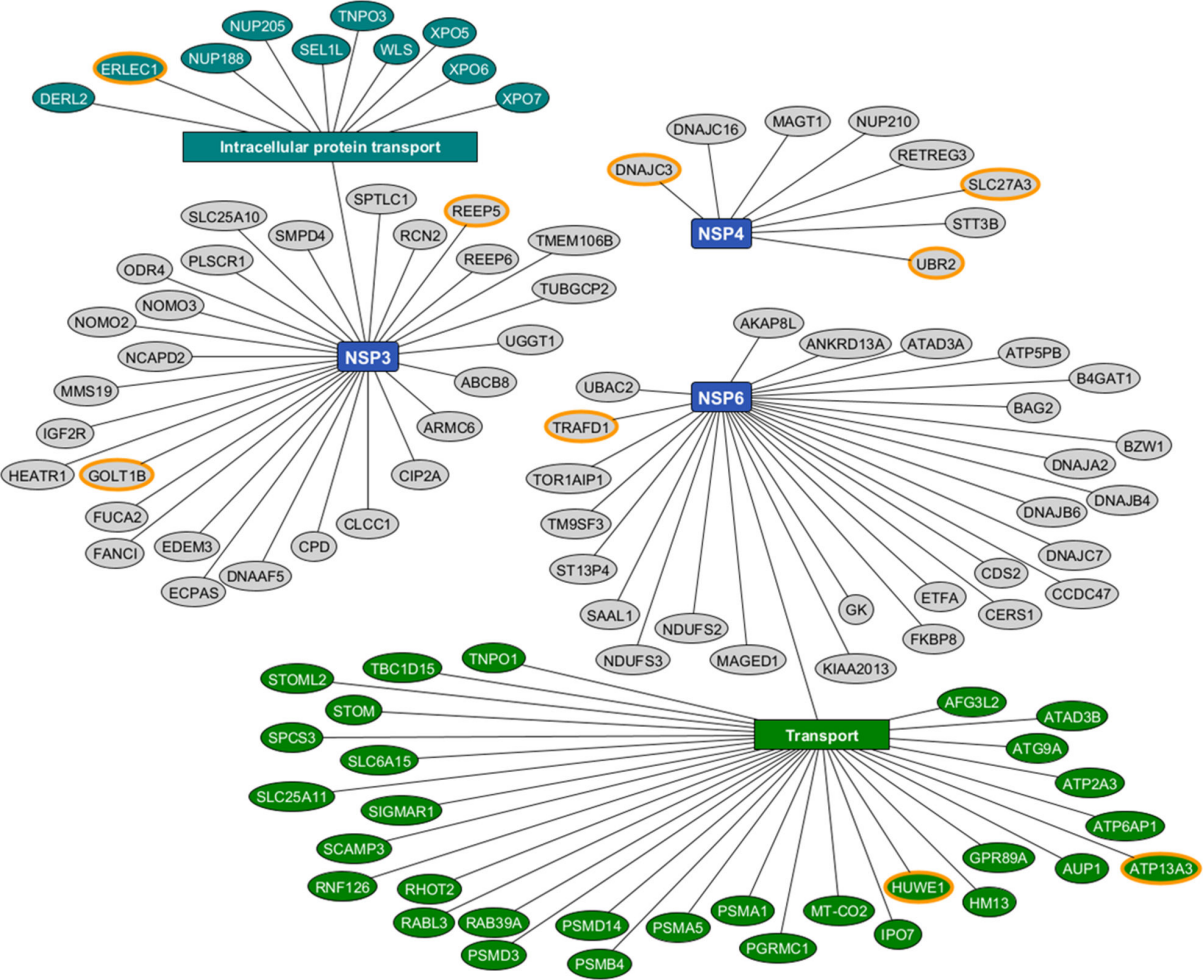
Host proteins binding SARS-CoV-2 NSP3C, NSP4, and NSP6 expressed individually. The graph shows 106 high-confidence host-binding proteins for SARS-CoV-2 NSP3C, NSP4, and NSP6 proteins (blue rectangles) individually. For host proteins binding more than one NSPs, we made graph to show their interaction to the NSP for which it had the highest enrichment. Enrichment analysis was done with the STRING website. Representative enrichments in the GO process category are shown in the plot (all enrichments were filtered at an false discovery rate (FDR) < 0.05 level). Proteins in the same biological process are categorized with teal and green ovals. Orange circles highlight the top three most specific host-binding proteins for each group.

To identify the specific host-binding proteins for each of the NSPs, we performed AP-Strep in HEK293T cells expressing 2Strep-tagged NSP3, NSP4, and NSP6 individually. The co-purified proteins from three AP-Strep biological replicates for each of the NSPs were analyzed by MS. Our AP-MS analysis identified 106 high-confidence PPIs between NSPs and host-binding proteins (Table S1). We define the high-confidence PPIs by (1) filtering out proteins with peptide-spectrum matches (PSMs) in any of the empty vector triplicates; (2) removing proteins with less than five PSMs on average per sample; (3) selecting proteins with a *t*-test *P*-value less than 0.01 as potential interactors of a given NSP AP-MS compared to the other two NSPs AP-MS. For host proteins binding more than one NSP, the graph only shows their interaction to the NSP for which it had the highest specificity score (Fig. 1). The top three most specific binding proteins for each of the NSP were highlighted with orange circles (NSP3C: REEP5, ERLEC1, and GOLT1B; NSP4: DNAJC3, SLC27A3, and UBR2; NSP6: TRAFD1, HUWE1, and ATP13A3). We analyzed each NSP for Gene Ontology enrichment with the STRING website (Table S1) and found that the representative cell process of the interacting proteins for NSP3C is intracellular protein transport (Fig. 1), in agreement with the essential role of NSP3 in RO biogenesis.

**Fig 2:**
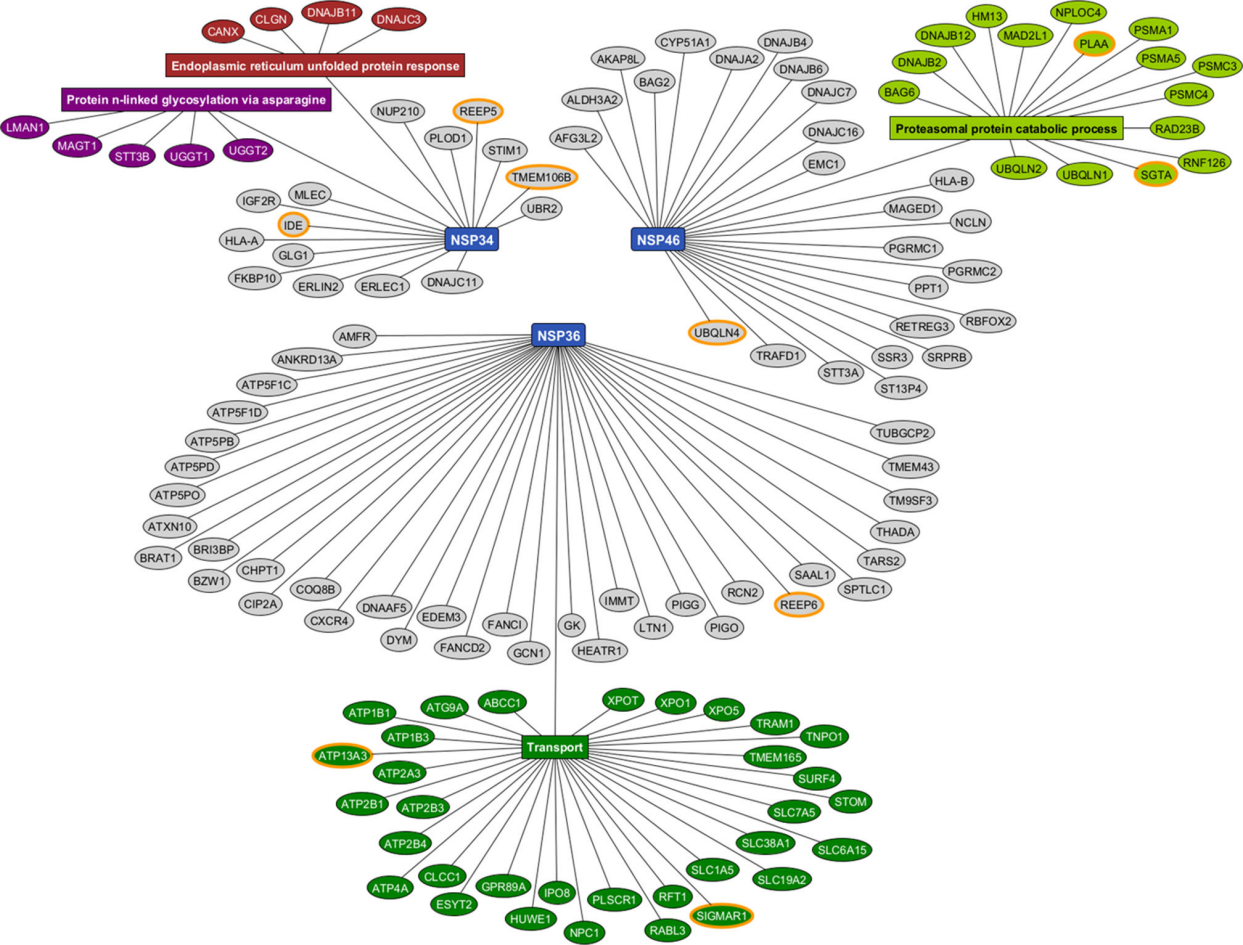
Host proteins binding SARS-CoV-2 NSP3C, NSP4, and NSP6 expressed in combination. The graph shows 135 high-confidence interactions between a combination of SARS-CoV-2 NSP3C, NSP4, or NSP6 proteins (NSP3+4, NSP4+6, and NSP3+6; blue rectangles) and human proteins. Each host protein is connected to the NSP for which it had the highest specificity. Enrichment analysis was done with the STRING website. Representative enrichments in the GO process category are shown in the plot (all enrichments were filtered at an FDR < 0.05 level). Proteins in the same biological process are categorized with red, purple, light, or dark green ovals. Orange circles highlight the top three most specific host-binding proteins for each group. The proteins labeled with * are also specific binding proteins of SARS-CoV-2 NSP3C, NSP4, and NSP6 individually, as shown in Figure 1.

Considering that viral proteins might combinationally interact with host proteins, we performed AP-MS in HEK293T cells expressing NSPs in different pairs (Table S1). We analyzed the AP-MS data of the pair of NSP3C and NSP4 (NSP3+4) with co-expression of NSP3C-2xStrep + NSP4 Flag, as well as NSP4-2xStrep + NSP3C-Flag; the pair of NSP4 and NSP6 (NSP4 +6) with co-expression of NSP4-2xStrep + NSP6 Flag, as well as NSP6-2xStrep + NSP4 Flag; the pair of NSP3C and NSP6 (NSP3 +6) with co-expression of NSP3C-2xStrep + NSP6 Flag, as well as NSP6-2Strep + NSP3C-Flag (Fig. 2). In total, we identified 135 high-confidence PPIs between each pair of NSPs and host-binding proteins, with 44 proteins (labeled with an asterisk) identified also in the experiments with individually expressed NSPs (Fig. 1). These results suggest that the study of NSP pairs is helpful to identify host-binding proteins compared to the analysis of individual NSPs. The top three most specific binding proteins for each of pair of NSPs were highlighted with orange circles (NSP3 + 4: REEP5, TMEM106B, and IDE; NSP4 + 6: PLAA, SGTA, and UBQLN4; NSP3 + 6: REEP6, ATP13A3, and SIGMAR1). After analyzing the host-binding proteins for Gene Ontology enrichment with the STRING website, we found that the representative cell processes of the interacting proteins for NSP3+4 are ER unfolded protein response and protein n-linked glycosylation via asparagine; for NSP4+6 is proteasomal protein catabolic process; for NSP3+6 is transport (Fig. 2).

### REEP5 and TRAM1 bind NSP3 at ROs

**Fig 3:**
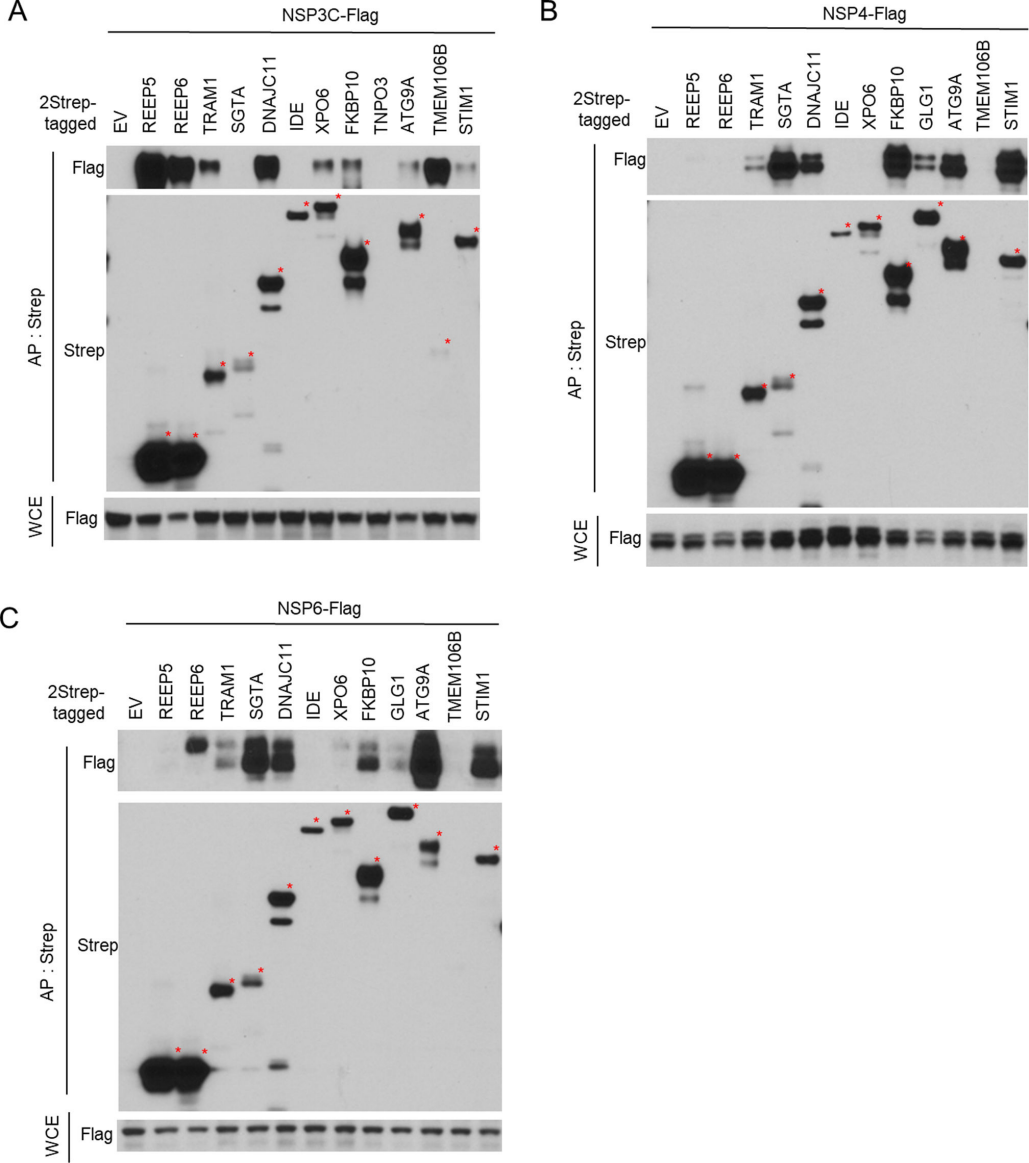
Validation of the binding proteins of SARS-CoV-2 NSP3C, NSP4, and NSP6. Immunoblot of AP-Strep from HEK293T cells co-transfected with Flag-tagged NSP3C (**A**), NSP4 (**B**), or NSP6 (**C**) and 2Strep-tagged plasmids as indicated. Each bait protein was marked with a red star. Whole cell extract (WCE) controls are shown at the bottom.

To identify the host-binding proteins of NSPs at ROs, we selected 13 candidates (REEP5, REEP6, TRAM1, SGTA, DNAJC11, IDE, XPO6, FKBP10, TNPO3, GLG1, ATG9A, TMEM106B, and STIM1) from the above AP-MS analysis based on protein localization at the ER and the reported relationship with virus replication. We cloned each of these candidate cDNAs into a mammalian expression vector with a 2xStrep affinity tag and co-expressed them with Flag-tagged NSP3C (Fig. 3A), NSP4 (Fig. 3B), or NSP6 (Fig. 3C), respectively. PPIs were detected by WB following AP-Strep. Except for TNPO3 (no detected expression) and IDE (expressed, but no detected binding with any NSPs), we confirmed that other 11 candidate proteins bind at least one NSP. Specifically, REEP5 and TMEM106B bind NSP3C; REEP6 binds NSP3C and NSP6; TRAM1, DNAJC11, FKBP10, ATG9A, and STIM1 bind NSP3C, NSP4, and NSP6; SGTA binds NSP4 and NSP6; and XPO6 binds NSP3C and NSP6. These data confirmed that our AP-MS analysis provided high-confidence information for viral-host PPIs.

**Fig 4:**
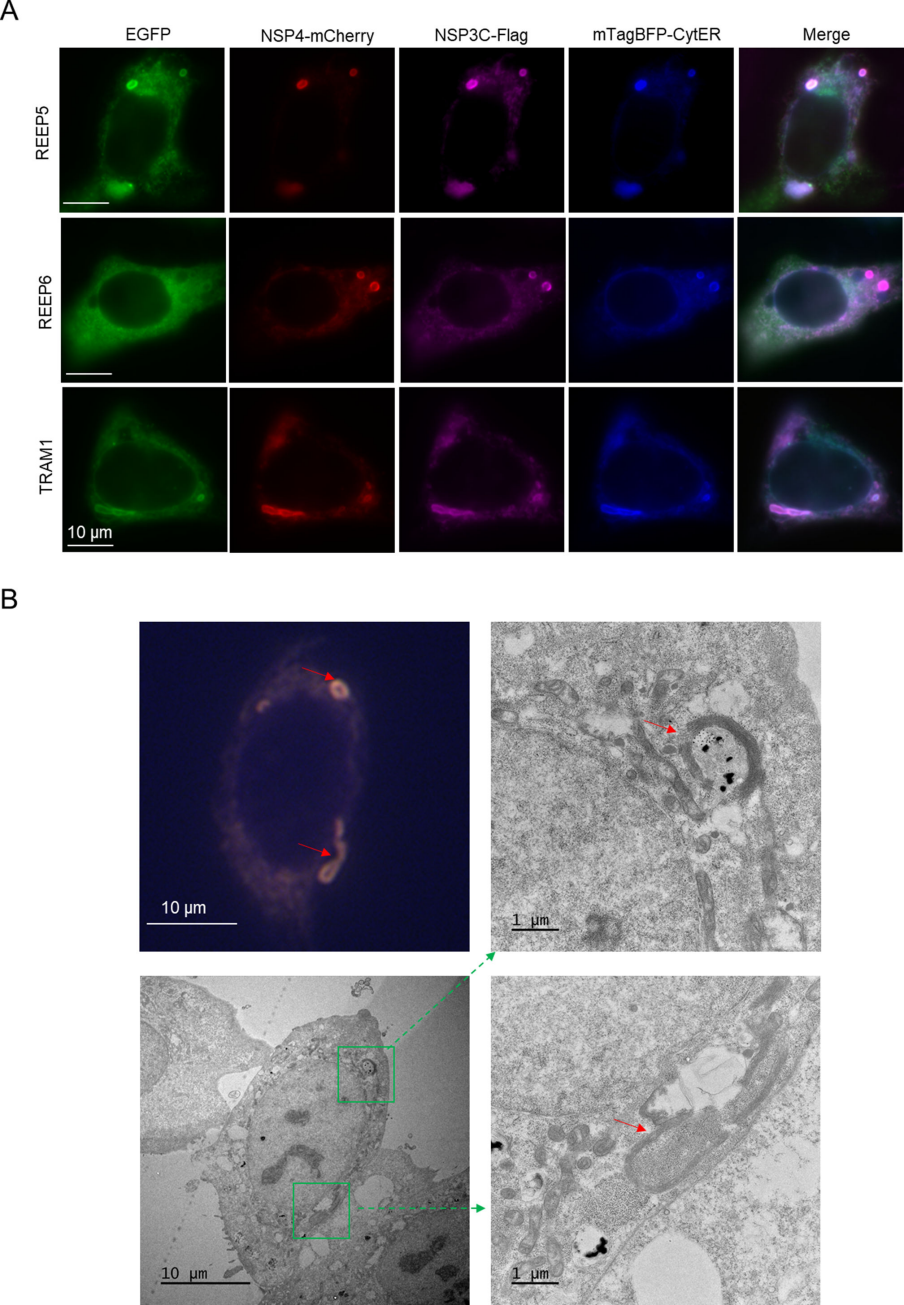
REEP5 and TRAM1 colocalize with SARS-CoV-2 NSP3 at double-membrane vesicles. (**A**) Representative fluorescence images from U-2 OS cells expressing NSP3C-Flag, NSP4-mCherry, and mTagBFP2-CytER were transfected with REEP5-EGFP, REEP6-EGFP, or TRAM1-EGFP. Scale bars: 10 µm. (**B**) Correlative light and electron microscopy of U-2 OS cells expressing REEP5-EGFP, NSP3C-mCherry, and mTagBFP2-CytER. Merging fluorescence images from live cells (Top left panel) and the matching electron microscopy image (bottom left panel) and amplification of areas inside the green square (right panel) are shown. MMVs are marked with red arrow. Scale bars: 10 µm (left panel) and 1 µm (right panel).

To pinpoint which host-binding proteins localize at ROs, we expressed these 11 validated host-binding proteins with an EGFP tag in U-2 OS cells expressing also Flag-tagged NSP3C, mCherry-tagged NSP4, and the mTagBFP2-tagged ER marker. Fluorescent images show that REEP5, TRAM1, and STIM1 co-localized at ROs with the ER marker, NSP3C, and NSP4 (Fig. 4A; Fig. S2). Moreover, we confirmed that the localization of REEP5 at ROs with CLEM (Fig. 4B).

**Fig 5:**
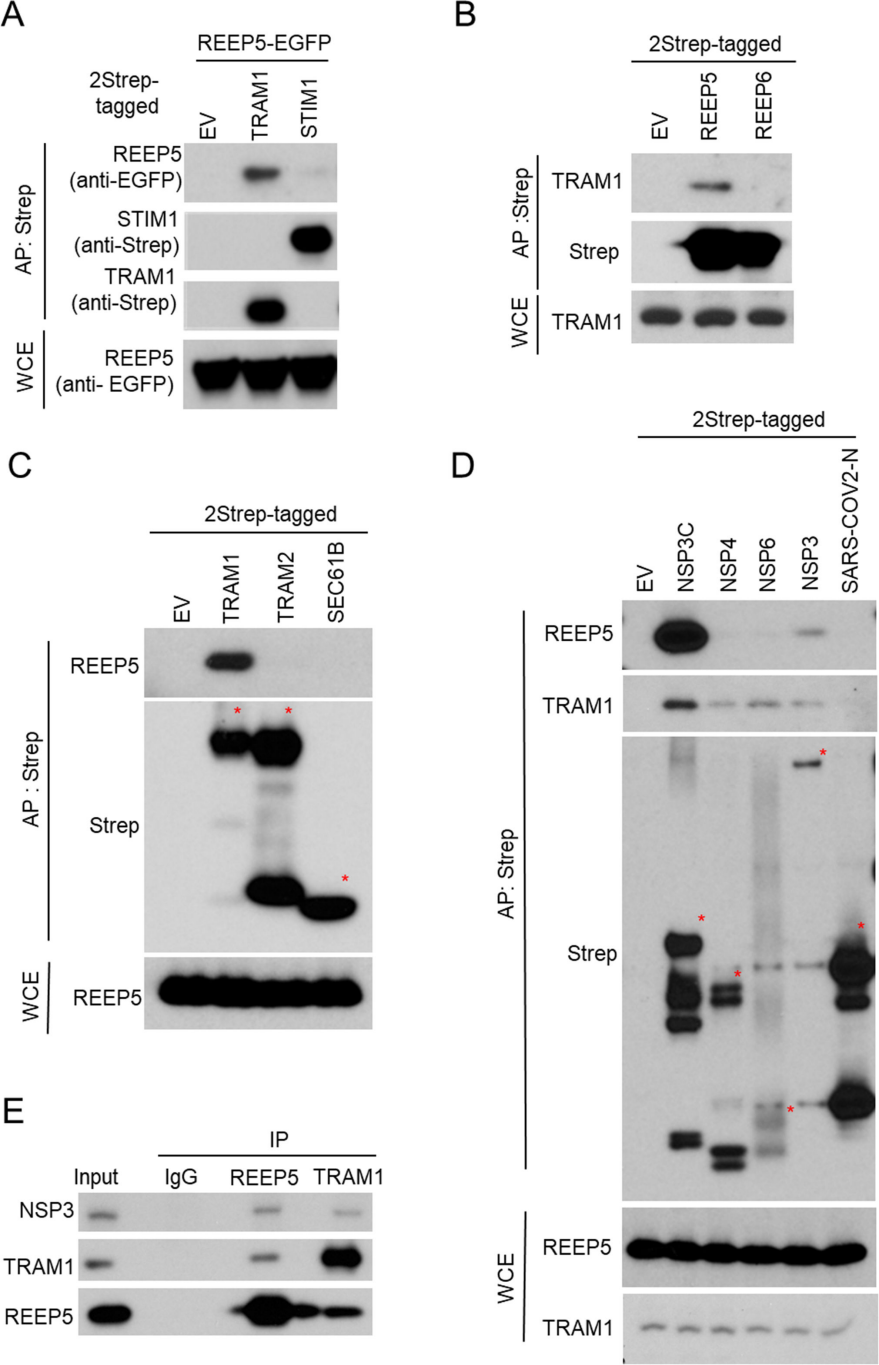
REEP5 and TRAM1 bind SARS-CoV-2 NSP3 during viral infection. (**A–D**) Immunoblot of AP-Strep from HEK293T cells co-transfected with indicated 2Strep-tagged plasmids as indicated. Each bait protein was marked with a red star (**C and D**). WCE controls are shown at the bottom. (**E**) Immunoblot of immunoprecipitation (IP) with indicated antibodies from Calu-3 cells after infection of SARS-CoV-2 (MOI = 5) for 24 hours.

Next, we asked whether REEP5, TRAM1, and STIM1 bind each other. We co-expressed EGFP-tagged REEP5 with 2xStrep-tagged TRAM1 or STIM1 in HEK293 cells and performed WB following AP-Strep (Fig. 5A). We found that REEP5 binds TRAM1 but not STIM1, suggesting that the REEP5/TRAM1 complex binds viral NSPs at ROs. Furthermore, we verified the binding specificity of REEP5/TRAM1 complex. Purification of 2xStrep-tagged REEP5, but not REEP6, pulled down endogenous TRAM1 (Fig. 5B). Similarly, purification of 2xStrep-tagged TRAM1, but not TRAM2 or SEC61B, pulled down endogenous REEP5 (Fig. 5C). In addition, purification of NSP3C or full-length NSP3 pulled down both endogenous REEP5 and TRAM1 (Fig. 5D). While TRAM1 also bound NSP4 and NSP6, REEP5 appeared to be a specific binding protein of NSP3 (Fig. 5D and Fig. 3). Importantly, we confirmed the endogenous binding between viral NSP3 and REEP5/TRAM1 complex in Calu-3 cells after infection with SARS-CoV-2 (Fig. 5E).

All of these data indicate the REEP5/TRAM1 complex as a bona fide host protein complex interacting with SARS-CoV-2 NSP3 at ROs.

### The REEP5/TRAM1 complex promotes ER membrane rearrangements and SARS-CoV-2 replication

**Fig 6:**
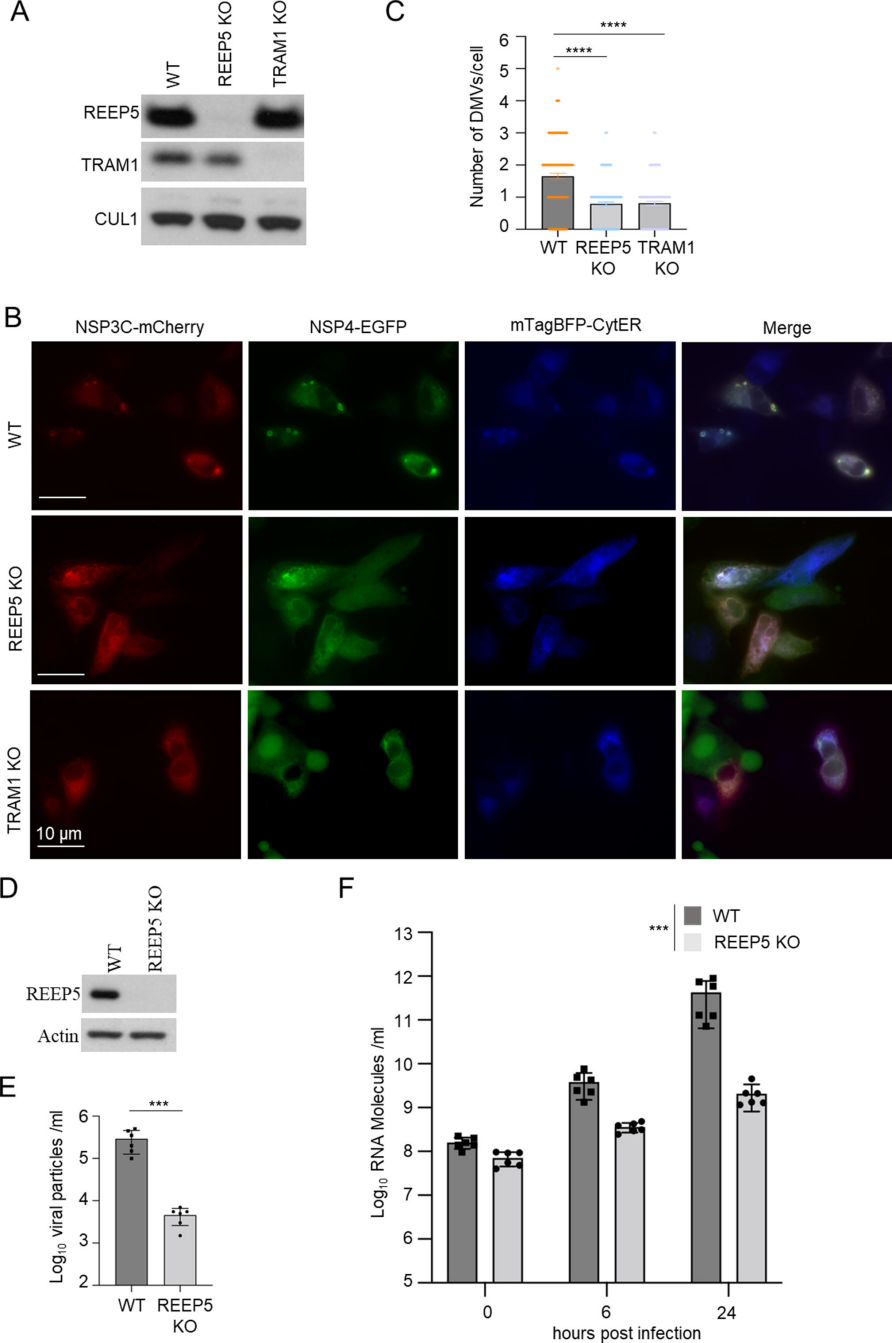
REEP5/TRAM1 complex promotes ER membrane rearrangements and SARS-CoV-2 replication. (**A**) The U-2 OS cells were infected with Cas9 and guide sgRNAs against a non-targeting control, REEP5 or TRAM1. Control and knockout (KO) cells were validated for REEP5/TRAM1 expression by WB. (**B**) Representative fluorescence images from WT, REEP5 KO, or TRAM1 KO U-2 OS cells were transfected with NSP3C-EGFP, NSP4-mCherry, and mTagBFP2-CytER. Scale bars: 10 µm. (**C**) Quantification of number of MMVs in Wild-type (WT), REEP5 KO, or TRAM1 KO U-2 OS cells. *N* > 150 cells for each cell lines from three independent experiments. Data are mean + SEM and individual values. Mann Whitney test was used. Differences were significant for ****P < 0.0001. (**D**) The Calu-3 cells were infected with Cas9 and guide sgRNAs against a non-targeting control or REEP5. WT and REEP5 KO Calu-3 cells were validated for REEP5/TRAM1 expression by WB. (**E**) Plaque-forming units (PFUs) determined by virus titration of supernatant from WT and REEP5 KO Calu-3 cells at 24 hours after infection with SARS-CoV-2 (MOI = 0.1). (**F**) RT-qPCR analysis of SARS-CoV-2 intracellular RNA expression WT and REEP5 KO Calu-3 cells upon 0, 6, and 24 hours after infection with SARS-CoV-2 (MOI = 0.1). For (**E**) and (**F**), *N* = 6 wells for each cell lines from three independent experiments. Data are mean + SEM and individual values. Mann Whitney test was used. Differences were significant for ***P < 0.001.

Since both REEP5 and TRAM1 localize at the ER and are important for ER membrane organization (24, 25), we asked whether the REEP5/TRAM1 complex regulates ER membrane rearrangements induced by co-expression of NSP3C and NSP4. U-2 OS cells were infected with lentivirus carrying either Cas9 and single guide RNAs (sgRNAs) against REEP5 or TRAM1 or a non-targeting control sgRNA. REEP5 knockout (KO) or TRAM1 KO U-2 OS cells were validated with immunoblot (Fig. 6A) and DNA sequencing (Fig. S3). In these cell lines, we co-expressed the EGFP-tagged NSP3C and mCherry-tagged NSP4 together with an mTagBFP2-tagged ER marker. Fluorescent images showed that the number of MMVs induced by NSP3C and NSP4 in REEP5 KO or TRAM1 KO cells is significantly lower compared to the number in parental cells (Fig. S6B and C). As ER membrane rearrangements are an essential step for the formation of MMVs, these data suggest that REEP5/TRAM1 complex plays a crucial role in SARS-CoV-2 RO biogenesis.

To further study the role of the REEP5/TRAM1 complex on SARS-CoV-2 replication, we used Calu-3 cells as a model because Calu-3 cells endogenously express the angiotensin-converting enzyme 2 (ACE2) receptor (26), a major cell entry receptor for SARS-CoV-2 (27). We infected Calu-3 cells with lentivirus carrying either Cas9 and a non-targeting control sgRNA, or guide RNAs (sgRNAs) against REEP5 or TRAM1. As the TRAM1 KO Calu-3 cells stopped dividing after infection, probably due to virus-induced cellular senescence, we validated REEP5 KO Calu-3 cells with immunoblot (Fig. 6D). We then infected the REEP5 KO and parental Calu-3 cells with SARS-CoV-2 for 24 hours and quantified plaque-forming units (PFU) by virus titration of supernatant from parental and REEP5 KO Calu-3 cells. The number of PFU in REEP5 KO is approximately 100 times lower compared to the number in parental cells (Fig. 6E). To measure viral RNA replication, we performed RT-qPCR analysis of SARS-CoV-2 RNA expression in REEP5 KO and WT Calu-3 cells at 0, 6, and 24 hours after infection with SARS-CoV-2. Depletion of REEP5 in Calu-3 cells also inhibited intracellular viral RNA level after SARS-CoV-2 infection for both 6 and 24 hours (Fig. 6F).

### REEP5 depletion reduces ER profile length and number of SARS-CoV-2-induced DMVs

**Fig 7:**
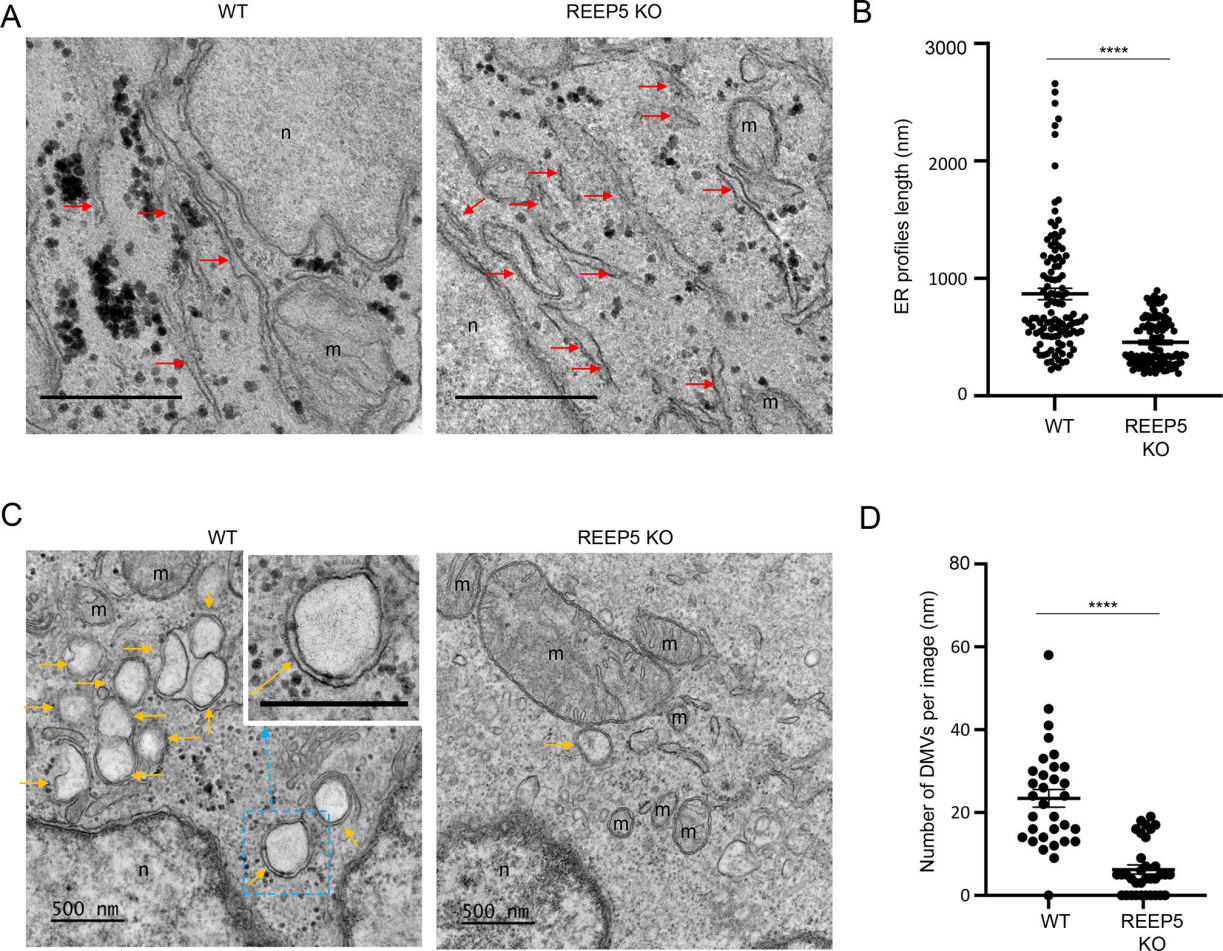
REEP5 depletion reduces ER profile length and SARS-CoV-2-induced DMVs. (**A**) Representative EM images of Caul-3 WT and REEP5 KO cells highlighting ER profiles with red arrows. Scale bar: 0.5 µm; m: mitochondria; n: nucleus. (**B**) Average length of ER profiles measured on EM pictures shown in (**A**), *n* > 100 ER profiles. (**C**) Representative EM images of Caul-3 WT and REEP KO cells highlighting DMVs with orange arrows. Scale bar: 0.5 µm; amplification: 10,000-fold; m: mitochondria; n: nucleus. The inset in the left panel shows a magnification of a DMV to highlight the two lipid bilayers characterizing these vesicles. (**D**) Average number of DMVs measured on EM pictures with the amplification of 2500-fold, *n* > 30 images. For (**B**) and (**D**), data are mean + SEM and individual values. Unpaired two-tailed *t*-test was used. Differences were significant for ****P < 0.0001. Error bars represent SEM.

To understand the role of REEP5 in ER morphology and SARS-CoV-2-induced DMVs, we infected parental and REEP5 KO Calu-3 cells with SARS-CoV-2 and compared their morphology at 0 (Fig. 7A and B) and 24 hours (Fig. 7C and D) after infection using electron microscopy. Consistent with a previous study (24), we observed the reduction of ER profiles length in REEP5 KO cells (Fig. 7A and B), suggesting instability in ER’s structure and function, which could lead to unfolded protein response, ER stress, ER-associated degradation (28), and autophagy (29). Similar to previous reports about virus-induced DMVs (3, 30), we observed DMVs with an average diameter of 150–350 nm, which were often clustered together in both parental and REEP5 KO Caul-3 cells at 24 hours after infection (Fig. 7C). Moreover, both the number (Fig. 7D) and size (Table 1) of SARS-CoV-2-induced DMVs were significantly decreased in REEP5 KO cells.

**TABLE 1:** Size of DMVs in infected WT and REEP5 KO Calu-3 cells

	No. of cells	No. of DMV	DMV (%)	Size (nm)	*P*
WT	32	750	96.88	319.30 ± 47.38	<0.0001
REEP5 KO	34	213	70.59	267.01 ± 34.21	

## DISCUSSION

Our work identifies a previously unknown REEP5/TRAM1 protein complex and its role on SARS-CoV-2 replication. Specifically, we provide a comprehensive host-viral protein-protein interactome for NSP3, NSP4, and NSP6 both individually and in combination. From the list of host-binding proteins, we found that two ER transmembrane proteins, REEP5 and TRAM1, bind each other, as well as NSP3 during SARS-CoV-2 infection. REEP5/TRAM1 complex colocalize with NSP3 and NSP4 at ROs and promote ER membrane rearrangement, thus promoting SARS-CoV-2 replication.

Despite accumulating studies on SARS-CoV-2 replication in recent years, knowledge on the critical host proteins involved in DMV biology is still relatively limited (9). We confirmed that endogenous REEP5 and TRAM1 bind NSP3 derived from SARS-CoV-2 infection. REEP5 belongs to a family of membrane curvature-stabilizing proteins, which contain a reticulon-homology domain that is thought to be essential for promoting and stabilizing curvature in ER tubules (31, 32). Consistent with a previous study (24), we observed the reduction of ER profiles length in REEP5 KO cells, suggesting instability in ER’s structure and function, which could induce unfolded protein response, ER stress, ER-associated degradation (28), and autophagy (29). As ER functions are exploited by SARS-CoV-2 to support distinct stages of their life cycle (33), it is possible that NSP3 binds REEP5 and disrupts its normal function, thus promoting ER membrane rearrangement and RO biogenesis. TRAM1 is instead an eight-transmembrane domain ER protein that is supposed to bind ceramide or related sphingolipids (34). TRAM1 was originally discovered as a component of the mammalian ER involved in the translocation of secretory proteins (35). Furthermore, TRAM1 was found as a protein interacting with some nascent membrane proteins during their initial integration into the Sec61-channel (36, 37). Since the Sec61-channel could translocate one-third of all polypeptides into or through the ER-membrane, TRAM1 may modulate the phospholipid bilayer near the lateral gate of the Sec61-channel to support ER protein translocation (38, 39). Although further validation is clearly required, TRAM1 is a potential host component of the pore complex at DMVs, suggesting that synthesized proteins required for viral RNA replication may be transported inside the DMVs via the pore complex.

Besides REEP5 and TRAM1, we confirmed eight other host-binding proteins, including TMEM106B, REEP6, DNAJC11, FKBP10, ATG9A, SGTA, XPO6, and STIM1. Intriguingly, in addition to be an NSP3 binding protein (Fig. 3A), TMEM106B has been recently reported to be an alternative receptor for SARS-CoV-2 entry into ACE2-negative cells (40). STIM1 colocalizes with NSP3C and NSP4 at ROs (Fig. S4A), and it is known to promote SARS-CoV-2 infection by decreasing type I interferon response (41). These findings may provide clues to study the mechanism of SARS-CoV-2 infection and immune evasion. Interestingly, the ER-resident proteins VMP1 and TMEM41B were identified as host proteins required for SARS-CoV-2 infection by genetic screens (42). A follow-up study showed that VMP1 and TMEM41B are involved in regulating DMV formation (10). While both NSP3 and NSP4 have the ability to bind VMP1, TMEM41B has a weak binding to NSP4, but not NSP3 (10). It is important to note that neither VMP1 nor TMEM41B was identified as high-confidence binding proteins of NSPs in our proteomics study (Table S1). This discrepancy could be because previous studies were conducted with overexpression of both “bait” and “prey” proteins as opposed to identifying endogenous binding partners.

The membrane phenotype induced by co-expression of NSP3C and NSP4 proteins does not fully resemble the DMVs biogenesis of SARS-CoV-2 infection. Besides lacking viral RNA synthesis, the abundance and morphology of these ROs induced by viral proteins are different from that of native DMVs during virus infection (9). This indicates that additional viral factors may play crucial roles on DMVs biogenesis. Gaining insight into this process from a molecular perspective will be essential to understand SARS-CoV-2 life cycle.

## MATERIALS AND METHODS

### Cell culture and treatments

HEK293T, U-2 OS, Calu-3, and Vero E6 cells were cultured in Dulbecco’s modified Eagle’s medium (DMEM) or Eagle’s Minimum Essential Medium (EMEM), supplemented with 10% fetal bovine serum (FBS) Penicillin/Streptomycin/L-Glutamine. Starvation experiments were performed after washing cells several times with phosphate-buffered saline (PBS) and adding either EBSS (Gibco) or DMEM without FBS supplementation. HEK293T and U-2 OS cells were transfected with plasmids with polyethylenimine. Lentivirus carrying Cas9 and guide sgRNAs were packaged in HEK293T cells.

To establish REEP5 KO and TRAM1 KO cell lines, U-2 OS or Calu-3 cells were infected with lentiviruses carrying Edit-R predesigned All-in-one lentiviral sgREEP5 or sgTRAM1 (Horizon Discovery), then selected with EGFP. KO cells were genotyped by extracting genomic DNA using DNeasy Blood and Tissue Kit (Qiagen), sgRNA targeting regions were amplified using Q5 polymerase (NEB) with the following primers, and sequences were analyzed with MacVector 18.6.0. REEP5 (forward), CTTGTCCCGTCTGTCTCCGA; REEP5 (reverse), GAGAGGTTCGACCGGTTCCT; TRAM1 (forward), GACTTTGCATCTCCGGGCC; TRAM1 (reverse), CCAGTGCTGAGCCACGAATT.

### Reagents

Sources of chemicals are found in the Key Reagents Table.

### Antibodies

The dilutions and sources of antibodies used for immunoblot (IB) and immunoprecipitation (IP) in this study can be found in the Key Resources Table. All antibodies used were validated following the multiple dilution method and, where available, using cell lines or tissues from animals knock-out for the antigen.

### Protein electrophoresis and immunoblotting

Protein concentration was determined using the Lowry method (43) with bovine serum albumin as the standard. Immunoblotting was performed after transferring SDS-PAGE gels to nitrocellulose membrane and blocking with 5% milk in 0.01% Tween-TBS for 1 hour at room temperature. The proteins of interest were visualized after incubation with primaries by chemiluminescence using horseradish peroxidase-conjugated secondary antibodies in the SRX-101A Tabletop X-Ray Film Processor (Konica).

### Affinity purification

HEK293T cells were transiently transfected with DNA using polyethylenimine (Polysciences). After transfection for 24 hours, cell lysis was carried out with lysis buffer (50 mM Tris pH 7.4, 150 mM NaCl, 10% glycerol, 0.3% Triton-X-100, and 0.1% NP-40) supplemented with protease and phosphatase inhibitors. Lysates were then immunoprecipitated with anti-FLAG antibody conjugated to agarose. After washing with lysis buffer for four times, elution was carried out with 3X FLAG peptide. For endogenous IP, lysates were incubated with anti-REEP5 or anti-TRAM1 antibody and rotated for 3 hours at 4°C. Then protein G beads were added and incubated for 1 hour at 4°C. After washing with lysis buffer for four times, the beads were denatured with 1X LDS for 3 min at 95°C. For affinity purification with MagStrep “type3” XT beads, elution was performed with Strep-Tactin XT elution buffer. For denaturing IP, cells were lysed with 2% SDS and denatured for 5 min at 95°C, then the lysates were diluted 1:20 to perform affinity purification.

### Fluorescent microscopy

Cells were plated on No.1.5 coverslip or glass bottom dish. Discard cell medium and add 2 mL of prewarmed fixative containing 2% paraformaldehyde and 0.1% glutaraldehyde in PBS for 15 min at room temperature; wash with PBS, 3 × 5 min each. To eliminate unbound aldehydes, cells were incubated in 50 mM glycine (37.5 mg in 10 mL) in PBS for 5 min (RT). Cells were permeabilized by 0.1% Triton X-100 in PBS for 10 min (RT); blocking with blocking solution (1% bovine serum albumin in PBS) for 60 min at RT on the shaker. Incubate with antibody in primary antibody incubation buffer (1% BSA in PBS) for 2 hours at RT on the shaker. Wash with PBS, 3 × 10 min, at RT on the shaker. Incubate with secondary antibody in dark in antibody incubation buffer (1% BSA in PBS) for 30 min to 1 hour, at RT on the shaker; wash with PBS, 3 × 10 min; wash/incubate with PBS/4',6-diamidino-2-phenylindole DAPI; wash with PBS, twice; For imaging, on the day of data collection, cells were incubated in PBS. Imaging was performed using Zeiss AxioObservor microscope.

### Correlative light and electron microscopy

For morphological analysis of autophagic vesicles, cultured cells were fixed in 0.1M sodium cacodylate buffer (pH 7.4) containing 2.5% glutaraldehyde and 2% paraformaldehyde overnight at 4°C and post-fixed with 1% osmium tetroxide mixed with 1% potassium ferrocyanide for 1 hour at 4°C, then block stained in 0.25% aqueous uranyl acetate overnight at 4°C, processed in a standard manner and embedded in EMbed 812 (Electron Microscopy Sciences, Hatfield, PA). Ultrathin sections (70 nm) were cut and mounted on 200 mesh copper grids. Quantitative analyses of ER profiles length were performed with ImageJ software using Feret’s Diameter (44).

For CLEM, cells were plated on gridded glass-bottom dishes (P35G-1.5–14-CGRD, MatTek) and fixed with 4% paraformaldehyde in 0.1 M sodium phosphate buffer (PBS) for 30 min at room temperature, then change to 2% paraformaldehyde in PBS and stored at 4°C overnight. Fluorescent and phase contrast images were taken at the areas of interest using Zeiss AxioObservor microscope. After light microscopy imaging, the cells are continue fixed with 2.5% glutaraldehyde for 1 hour and post fixed with 1% osmium tetroxide for 1 hour at room temperature. The cells were then block stained with 1% uranyl acetate for 1 hour, dehydrated in ethanol, and en face embedded in Araldite 502 (Electron Microscopy Sciences, Hatfield, PA). En face serial thin sections with 80 nm were cut and mounted on formvar coated slot copper grids.

All EM grids were stained with uranyl acetate and lead citrate by standard methods and examined under either Philips CM-12 electron microscope (FEI; Eindhoven, The Netherlands) and photographed with a Gatan (4k x2.7k) digital camera, or Talos L120C electron microscope (Thermo Fisher Scientific, Hillsboro, OR) coupled with Gatan 4k × 4k OneView Camera (Gatan Inc. Pleasanton, CA).

### Mass spectrometry

Samples were reduced with DTT at 57°C for 1 hour (2 µL of 0.2 M). Samples were then alkylated with iodoacetamide at RT in the dark for 45 min (2 µL of 0.5 M) and loaded onto NuPAGE 4%–12% Bis-Tris Gel 1.0 mM (Life Technologies Corporation) and ran for approximately 2 min at 200 V. The gel was stained using GelCode Blue Stain Reagent (Thermo Scientific), and Coomassie-stained gel bands were excised as indicated on the gel image. Excised gel pieces were destained in 1:1 v/v solution of Methanol and 100 mM Ammonium Bicarbonate solution. The gel pieces were partially dehydrated with an acetonitrile rinse and further dried in a SpeedVac concentrator for 20 min. 200 ng of sequencing grade modified trypsin (Promega) was added to each gel sample. After the trypsin was absorbed, 250 µL of 100 mM ammonium bicarbonate was added to cover the gel pieces. Digestion proceeded overnight on a shaker at RT. A slurry of *R2* 20 µM Poros beads (Life Technologies Corporation) in 5% formic acid and 0.2% trifluoroacetic acid (TFA) was added to each sample at a volume equal to that of the ammonium bicarbonate added for digestion. The samples shook at 4°C for 3 hour. The beads were loaded onto equilibrated C18 ziptips (Millipore) using a microcentrifuge for 30 seconds at 6,000 rpm. Gel pieces were rinsed three times with 0.1% TFA, and each rinse was added to its corresponding ziptip followed by microcentrifugation. The extracted beads were further washed with 0.5% acetic acid. Peptides were eluted by the addition of 40% acetonitrile in 0.5% acetic acid followed by the addition of 80% acetonitrile in 0.5% acetic acid. The organic solvent was removed using a SpeedVac concentrator and the sample reconstituted in 0.5% acetic acid. Sample was analyzed individually using LC separation online with MS using the autosampler of an EASY-nLC 1000 (Thermo Scientific). Peptides were gradient eluted from the column directly to a Orbitrap Eclipse mass spectrometer using a 1 hour gradient (Thermo Scientific) Solvent A: 2% acetonitrile, 0.5% acetic acid; Solvent B: 90% acetonitrile, 0.5% acetic acid. High-resolution full MS spectra were acquired with a resolution of 240,000, an AGC target of 1e^6^, with a maximum ion time of 50 ms, and scan range of 400–1,500 m/z. Following each full MS, data-dependent low-resolution ion trap HCD MS/MS spectra were acquired. All MS/MS spectra were collected using the following instrument parameters: ion trap rapid scan, AGC target of 2e^4^, maximum ion time of 18 ms, one microscan, 0.7 m/z isolation window, 20 seconds dynamic exclusion, fixed first mass of 150 m/z, and NCE of 27. Singly charged ions and ions carrying eight or more charges were excluded from triggering an MS/MS scan. The instrument was set to acquire a full MS scan every 3 seconds or earlier if no new MS/MS precursors were detected.

The MS/MS spectra were searched against a Uniprot (www.uniprot.org) human protein database with common lab contaminants and the sequence of the tagged bait proteins added using Sequest within Proteome Discoverer 1.4 (Thermo Fisher). The search parameters were as follows: mass accuracy better than 10 ppm for MS1 and 0.02 Da for MS2, two missed cleavages, fixed modification carbamidomethyl on cysteine, variable modification of oxidation on methionine, and deamidation on asparagine and glutamine. The data were filtered using a 1% FDR cut off for peptides and proteins against a decoy database and only proteins with at least two unique peptides were reported.

For MS data analysis, the input that we used for STRING website is listed in Table S1. The specificity score was defined as the proportion of PSMs detected in a given NSP triplicate out of the total PSM for that protein in the experiment.

### Quantification and statistical analysis

All data presented are mean ± SEM and individual values. Prior to statistical testing, normality was assessed using the Shapiro Wilk test. Statistical significance was compared by two-tailed unpaired Student’s *t*-test for two groups, one-way ANOVA for a single parameter in multiple groups, or two-way ANOVA for multiple parameters in multiple groups. The post hoc test used for multiple comparisons is stated in the legend of the figures. Statistical analyses were performed in GraphPad Prism 9.0.

### Virus infection and amplification test

All SARS-CoV-2 work was conducted in the NYU Grossman School of Medicine Biosafety Level three facility. SARS-CoV-2 (USA-WA1/2020 – BEI Resources) was obtained from Dr. Mark Mulligan at the NYU Grossman School of Medicine. The virus was amplified once over Vero E6 cells to obtain a working stock. Viral titers were determined by plaque assay on Vero E6 cells. In brief, 10-fold virus dilutions were made in DMEM and incubated on a monolayer of Vero E6 cells for 1 hour at 37°C. Following incubation, an 0.8% agarose overlay in DMEM with 2% FBS was added, and the cells were incubated for 72 hours. The cells were then fixed with formalin, agarose plugs removed, and cells stained with crystal violet. Viral titers were determined by counting plaques on the lowest countable dilution.

For SARS-CoV-2 PPI studies, Calu3 cells were incubated for 24 hours at 37°C in the presence of SARS-CoV-2 (MOI = 5). After incubation, the inoculum was removed, cells washed three times with PBS and resuspended in lysis buffer for affinity purification. For SARS-CoV-2 infections in Calu 3 REEP5 knockout cells, cells were incubated with virus diluted in DMEM (MOI = 0.1) for 1 hour at 37°C. Following incubation, the inoculum was removed, cells were washed twice with PBS, and DMEM containing 2% FBS was added for the indicated time. At each time point, the virus-containing supernatant was removed, and viral titers were quantified by plaque assay. For RNA quantification, the cells were washed three times with PBS, harvested in Trizol, and RNA extracted using the manufacturer’s instructions. SARS-CoV-2 RNA was quantified by RT-qPCR using a Taqman RNA-to-Ct kit (Applied Biosystems) with the following primers to the SARS-CoV-2 N protein (Forward: 5’ ATGCTGCAATCGTGCTACAA 3’, Reverse: 5’ GACTGCCGCCTCTGCTC 3’, and Probe: 56-FAM/TCAAGGAAC/ZEN/AACATTGCCAA/3IABkFQ/). A SARS-CoV-2 N protein RNA was *in vitro* transcribed and used as a standard to quantify SARS-CoV-2 RNA. All RT-qPCR samples and standards were run in technical duplicates.

## SUPPLEMENTAL MATERIAL

Fig. S1 jvi.00507-23-s0001.docx Expression and function of SARS-CoV-2 NSP3, NSP4, and NSP6 proteins in mammalian cells.

Raw data table jvi.00507-23-s0002.xlsx Original data for the statistical analysis of Figures 6C, 6E–F, 7B, 7D, and Table 1.

Uncropped WB pictures jvi.00507-23-s0003.pdf Uncropped WB pictures for both main figures and supplemental figures.

Fig. S2 jvi.00507-23-s0004.docx Colocalization of host-binding proteins with NSP3C and NSP4 at ROs.

Fig. S3 jvi.00507-23-s0005.docx Validation of REEP5 and TRAM1 KO U-2 OS cells.

Supplemental material legend jvi.00507-23-s0006.docx Checklist of supplemental material

Additional experimental details jvi.00507-23-s0007.xlsx Key Reagents Table including the information of antibodies, chemical reagents, plasmids, primers, and cell lines.

Table S1 jvi.00507-23-s0008.xlsx Search results of MS analysis for proteins co-IPed from HEK293T cells transiently expressing NSPs proteins.

ASM does not own the copyrights to Supplemental Material that may be linked to, or accessed through, an article. The authors have granted ASM a non-exclusive, world-wide license to publish the Supplemental Material files. Please contact the corresponding author directly for reuse.
