# Supplementary material for: The REEP5/TRAM1 complex binds SARS-CoV-2 NSP3 and promotes virus replication
Source: J Virol. 2023 Sep 28;97(10):e00507-23. doi: 10.1128/jvi.00507-23 (PMC10617467; doi:10.1128/jvi.00507-23)
Supplement: Fig. S1 — Expression and function of SARS-CoV-2 NSP3, NSP4, and NSP6 proteins in mammalian cells. [file jvi.00507-23-s0001.docx]

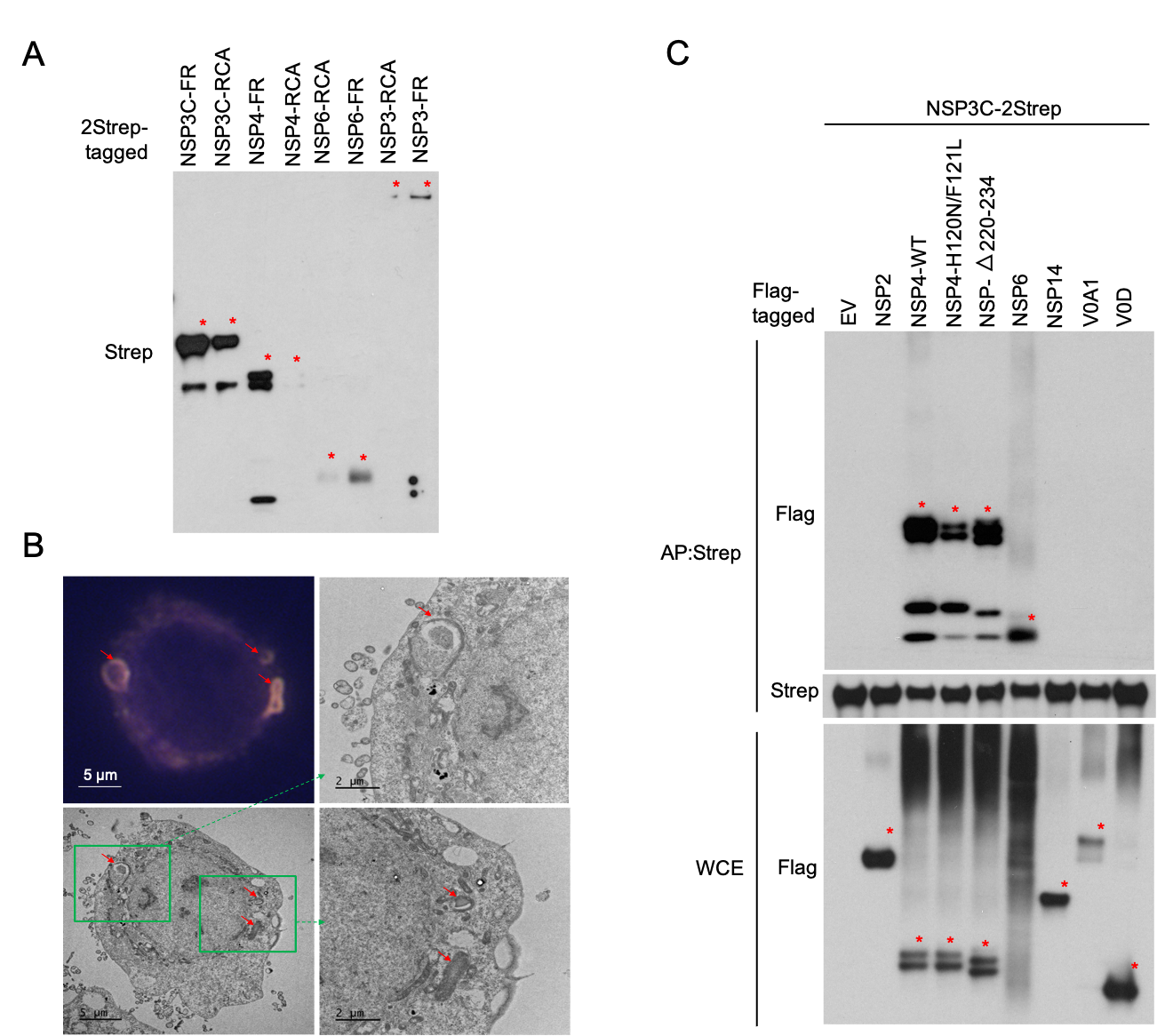


**Figure S1. Expression and function of SARS-CoV-2 NSP3, NSP4 and NSP6 proteins in mammalian cells.** (**A**)Immunoblot with anti-Strep on whole cell extract (WCE) from HEK293T cells transfected with plasmids as indicated. Each band corresponding to the predict molecular weight was marked with a red star. (**B**) Correlative light and electron microscopy of U-2 OS cells expressing NSP3C-EGFP, NSP4-mCherry and mTagBFP2-CytER. Merging fluorescence images from live cells (Top left panel) and the matching electron microcopy image (bottom left panel), and amplification of areas inside the green square (right panel) are shown. Multi-membrane vesicles (MMVs) are marked with red arrow. Scale bars: 5 μm (left panel) and 2 μm (right panel). (**C**) Immunoblot of affinity purification (AP) with Strep-tag (Strep) from HEK293T cells co-transfected with 2Strep -tagged NSP3C and Flag-tagged plasmids as indicated. Each band corresponding to the predict molecular weight was marked with a red star. Whole cell extract (WCE) controls are shown at the bottom.
