# Supplementary figures and images for: The REEP5/TRAM1 complex binds SARS-CoV-2 NSP3 and promotes virus replication
Source: J Virol. 2023 Sep 28;97(10):e00507-23. doi: 10.1128/jvi.00507-23 (PMC10617467; doi:10.1128/jvi.00507-23)

3A

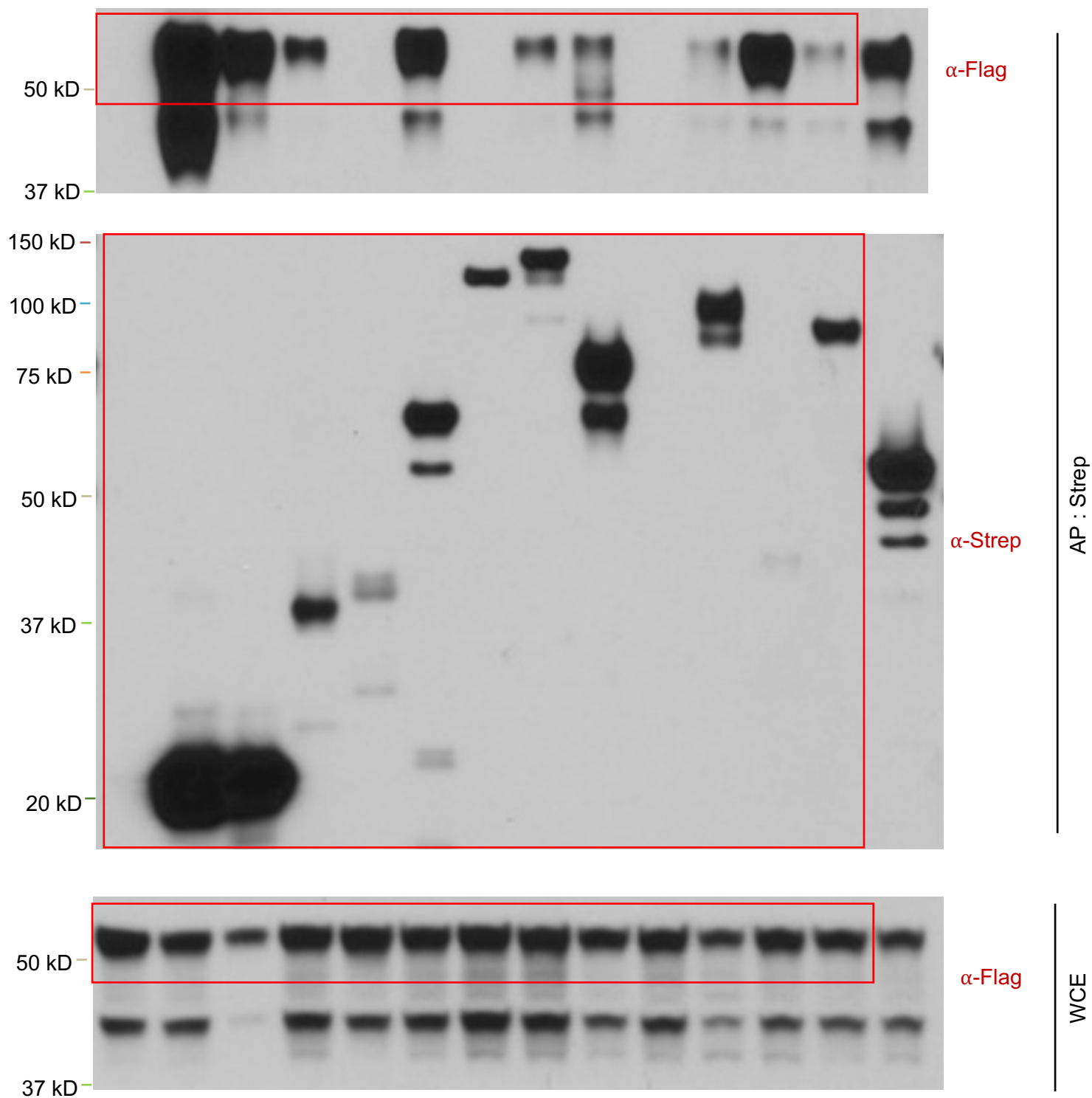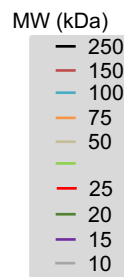

3B

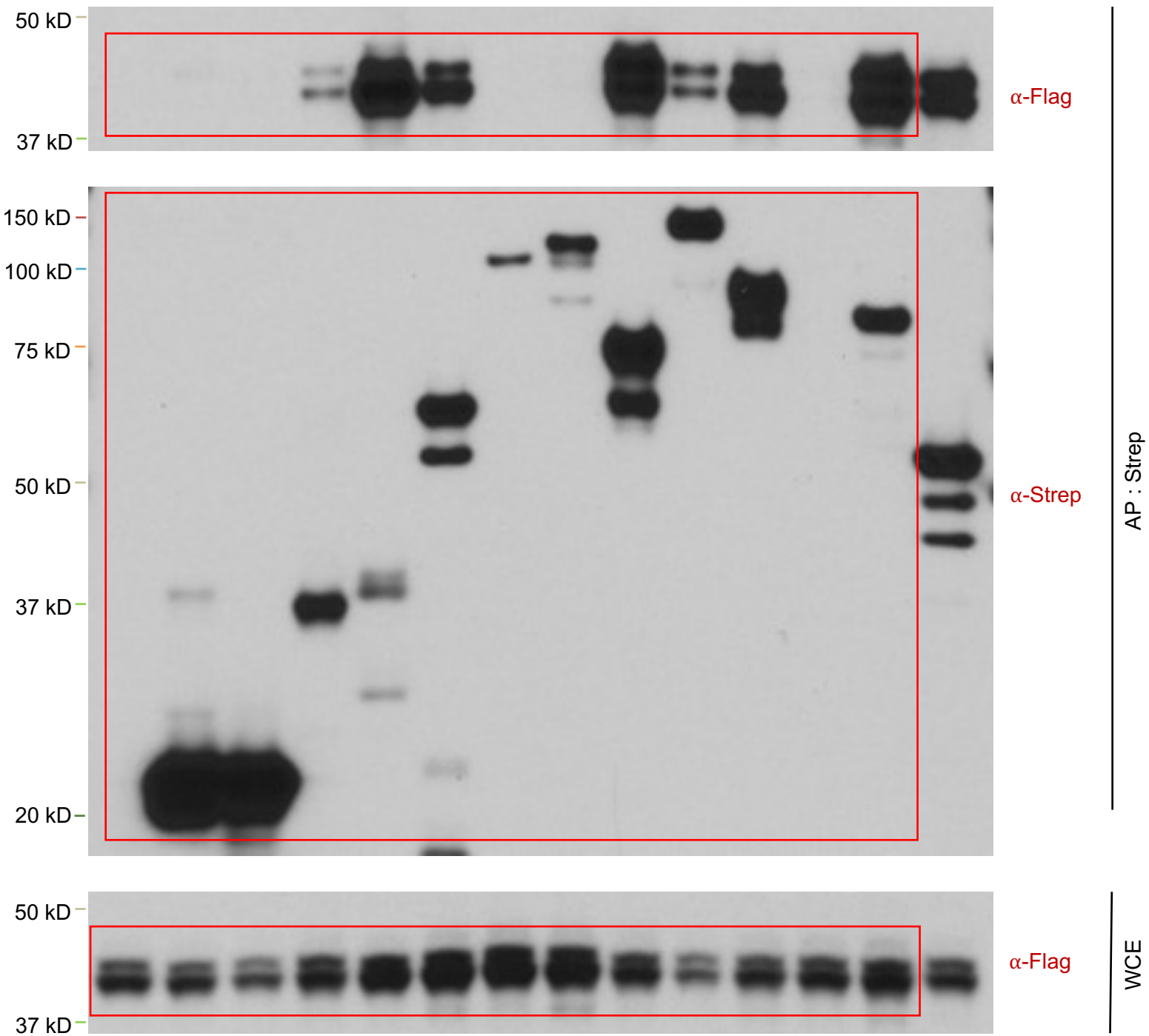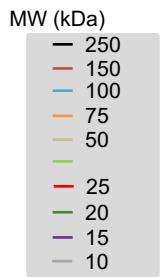

3C

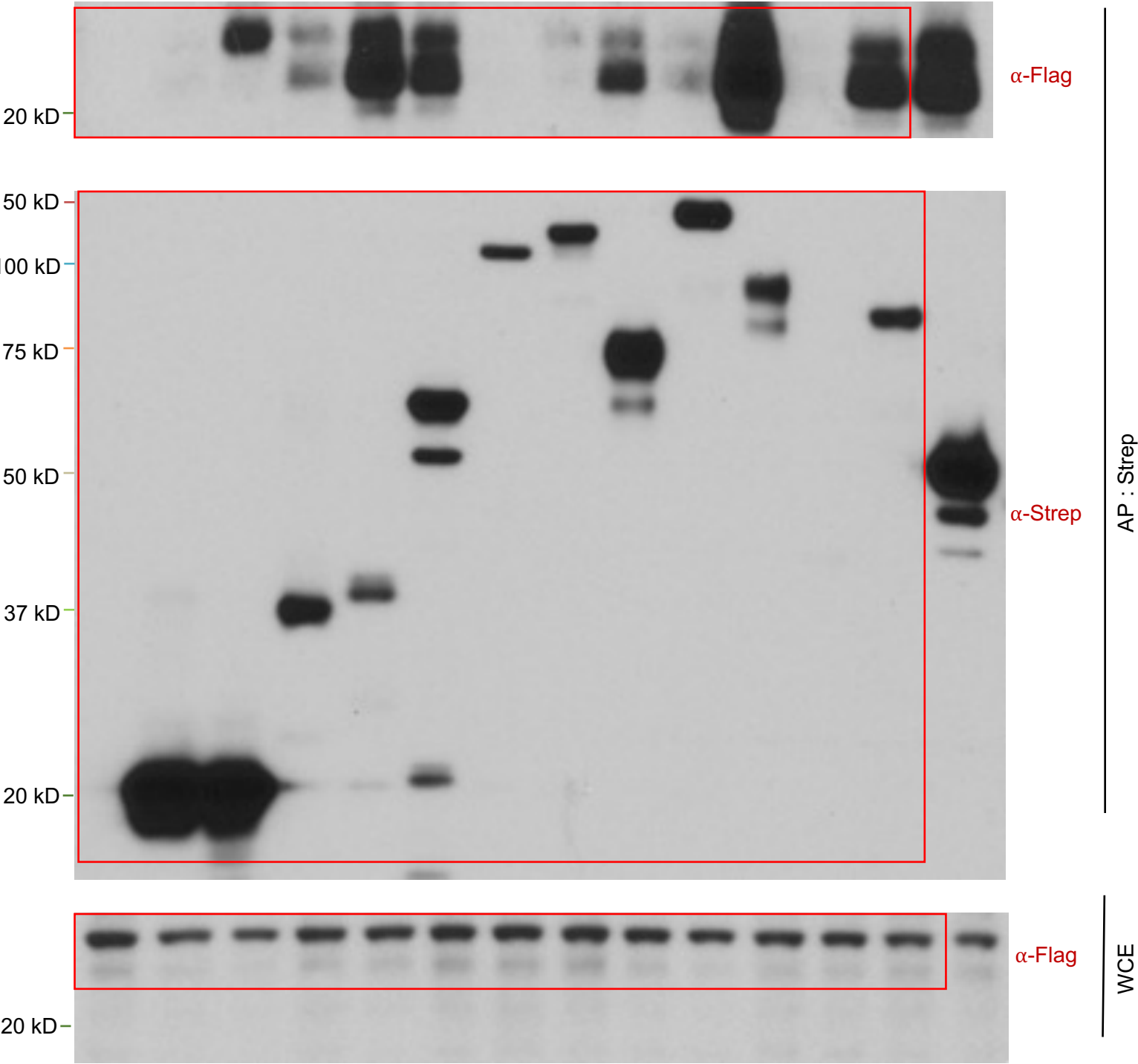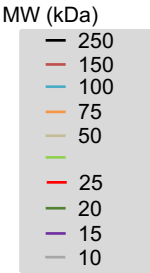

5A

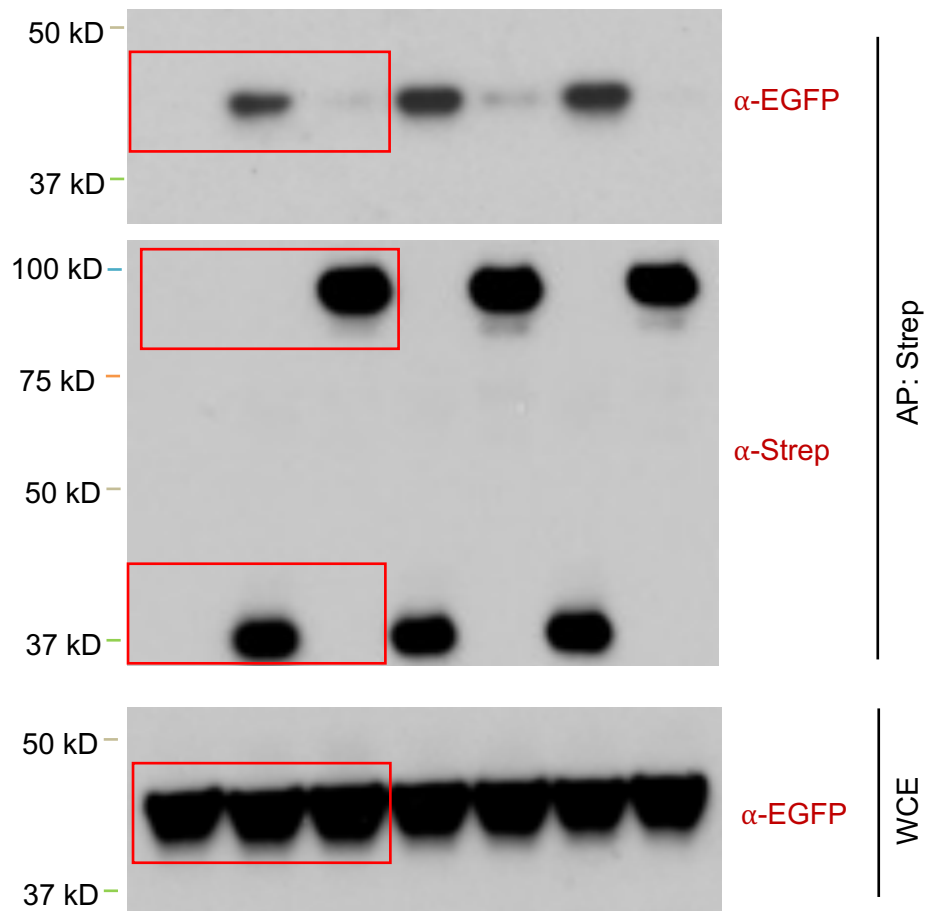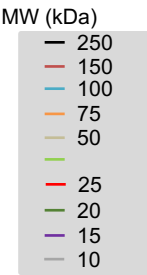

5B

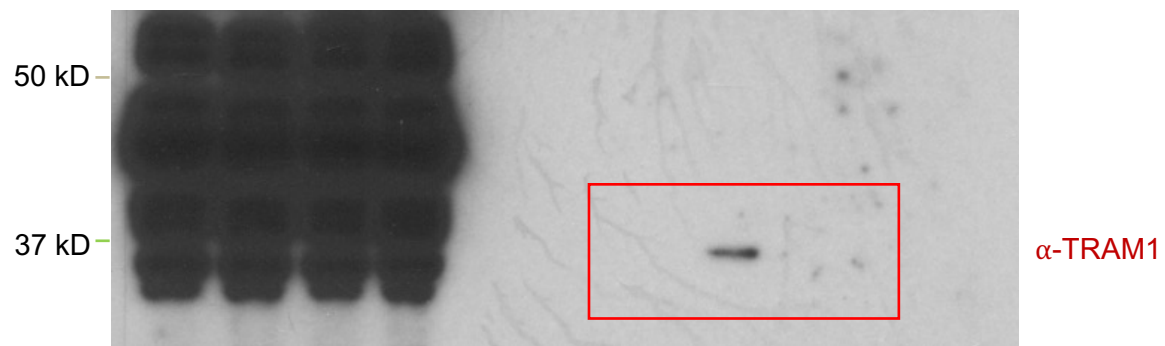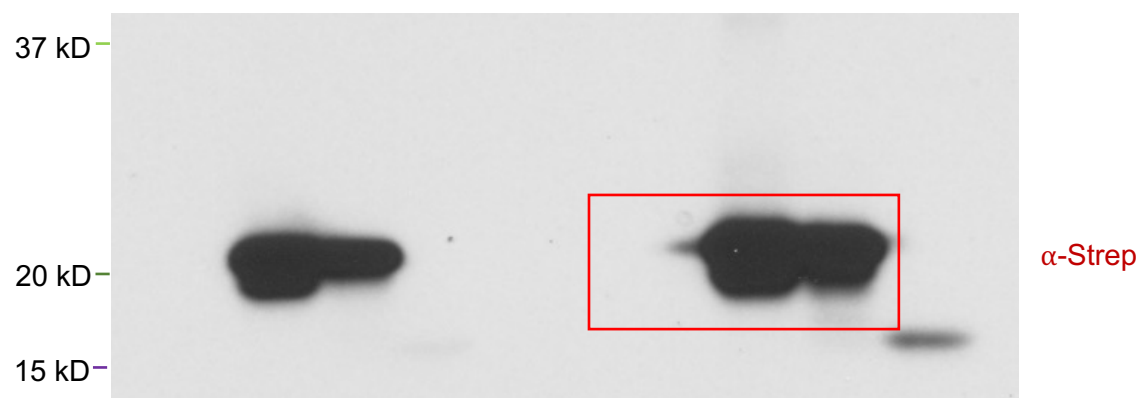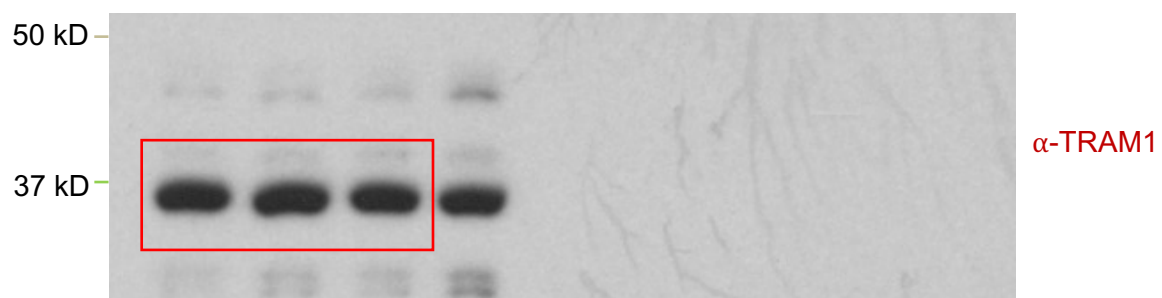

MW (kDa)

— 250  
— 150  
— 100  
— 75  
— 50  
— 25  
— 20  
— 15  
— 10

5C

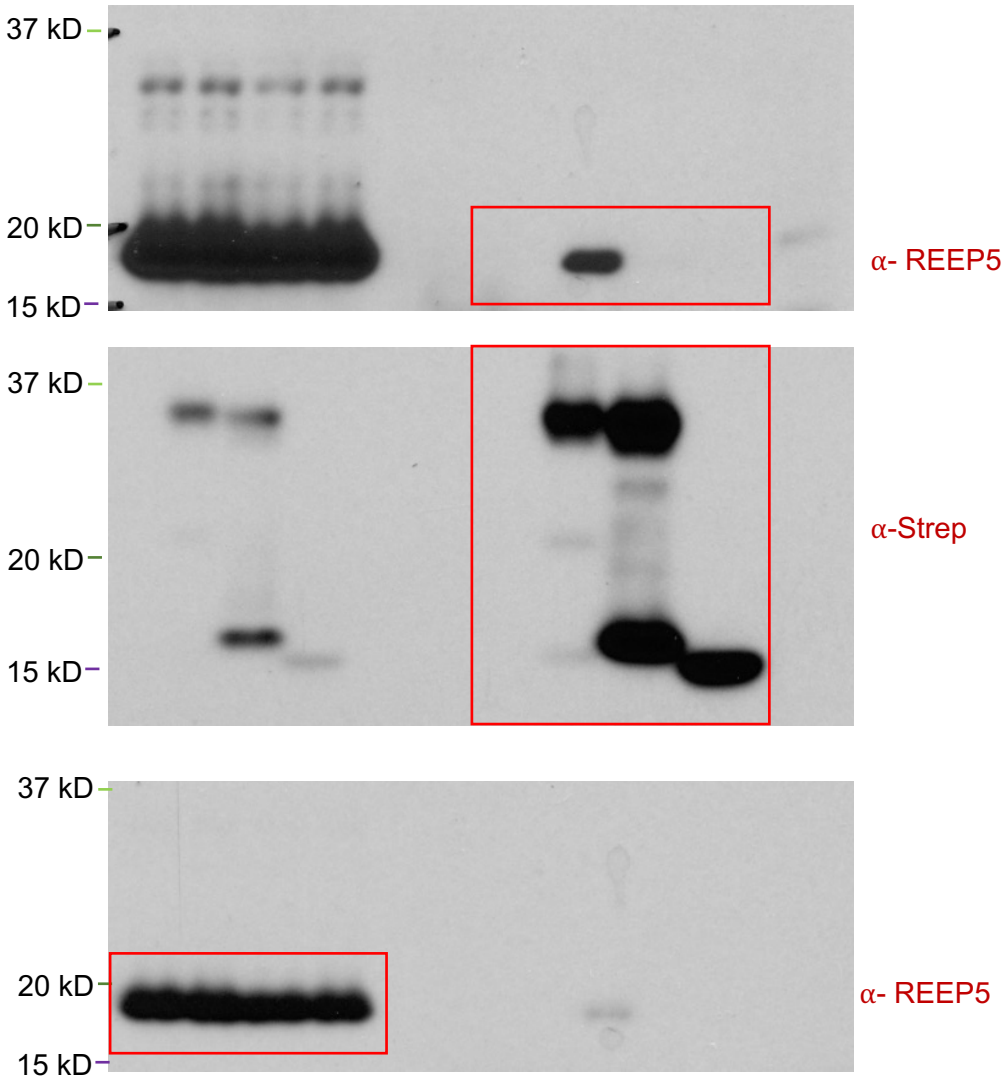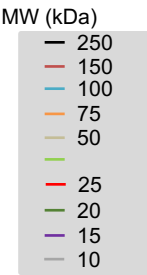

5D

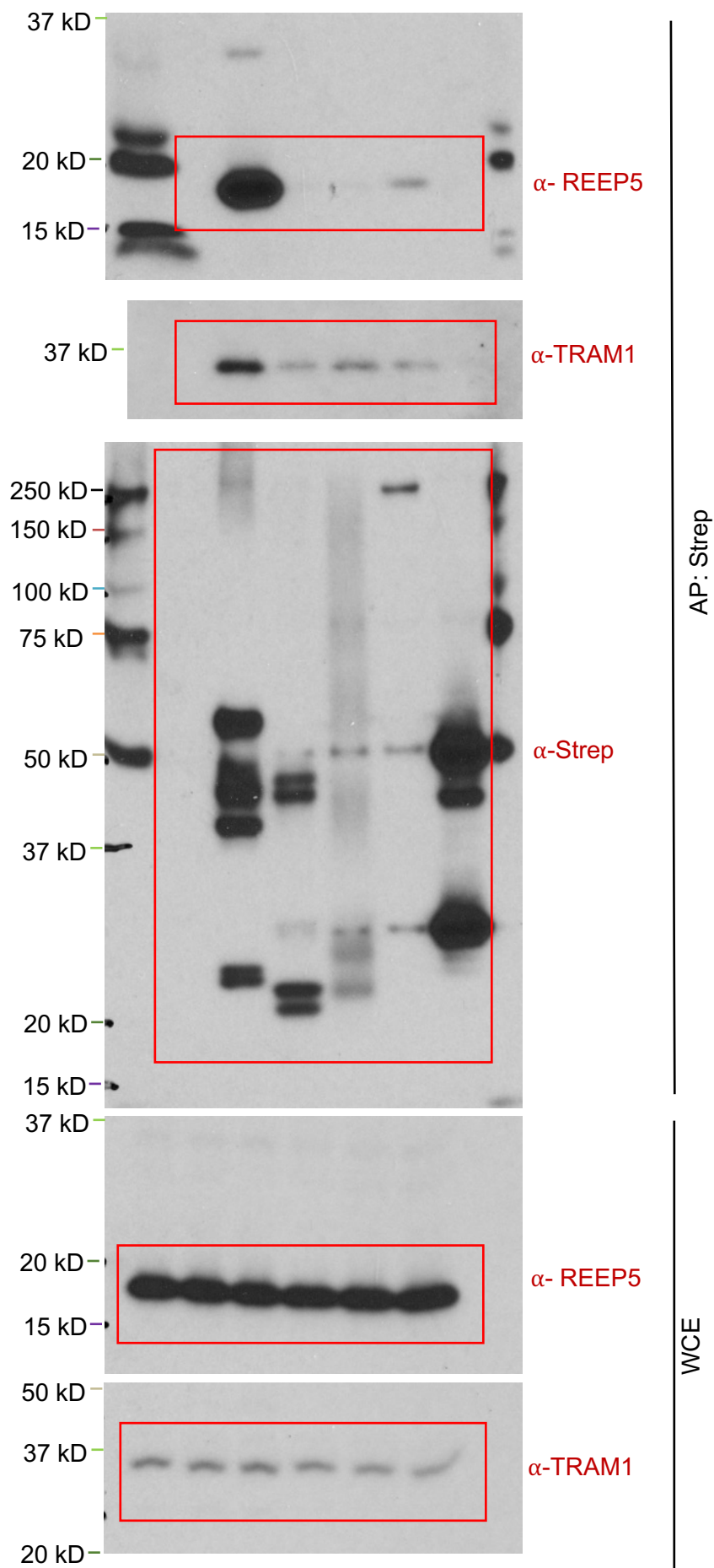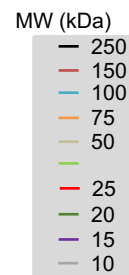

5E

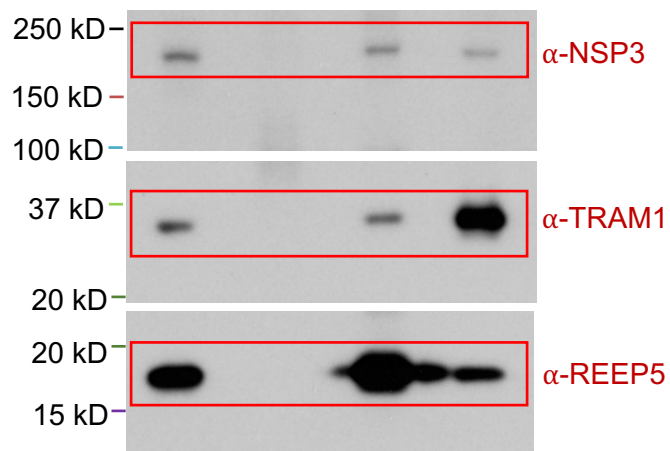

6A

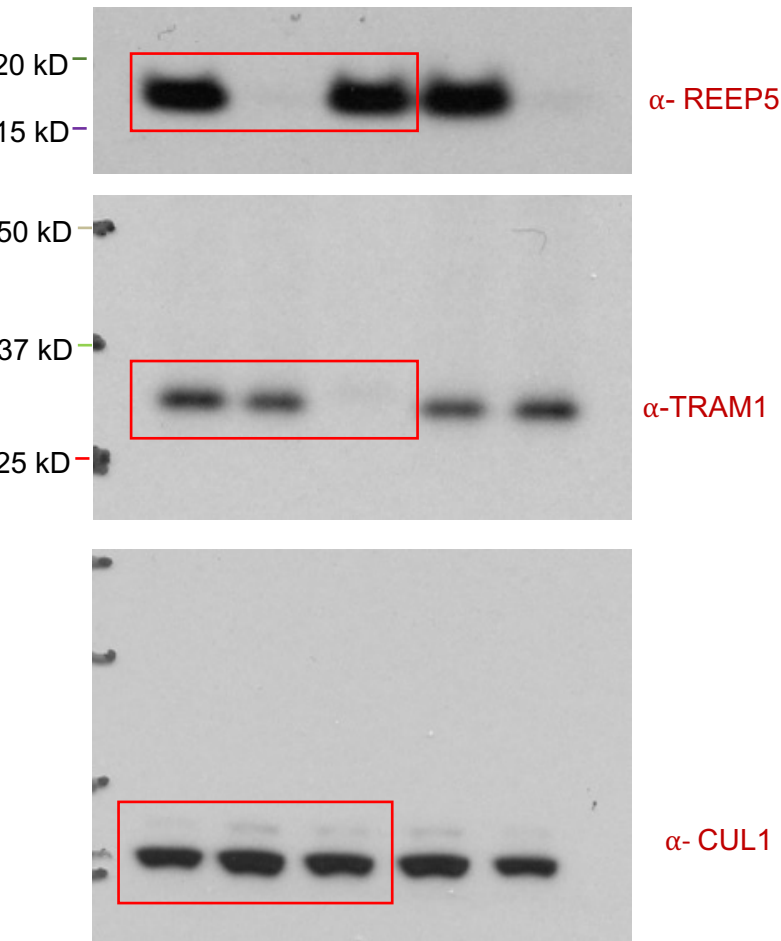

6D

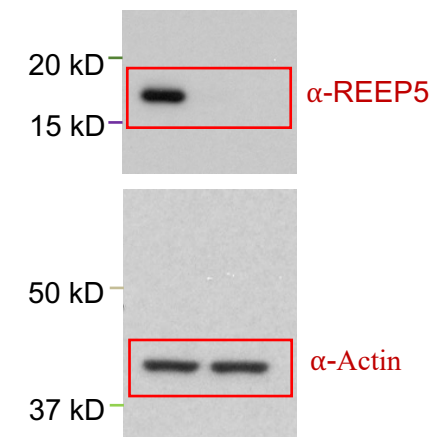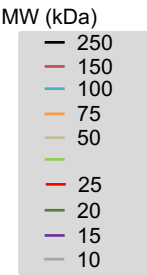

S1A

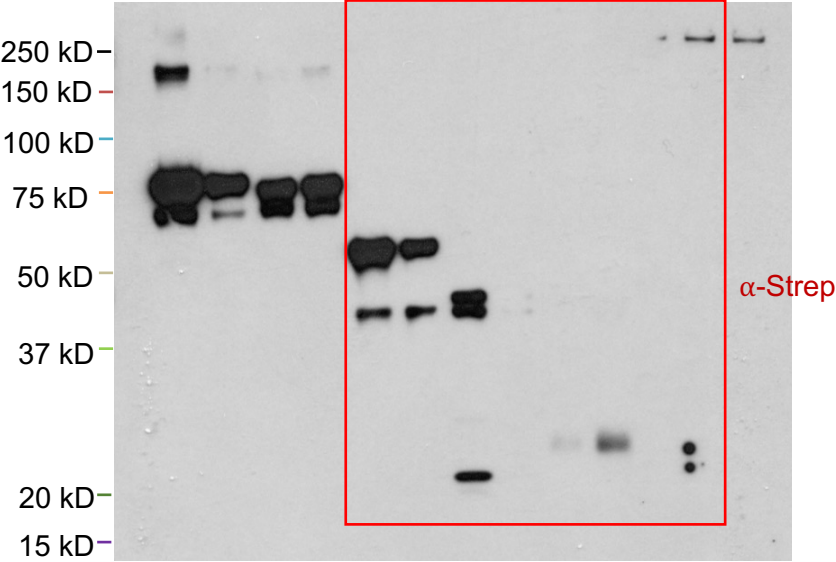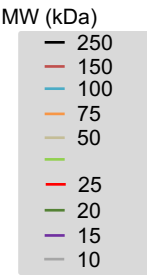

S1C

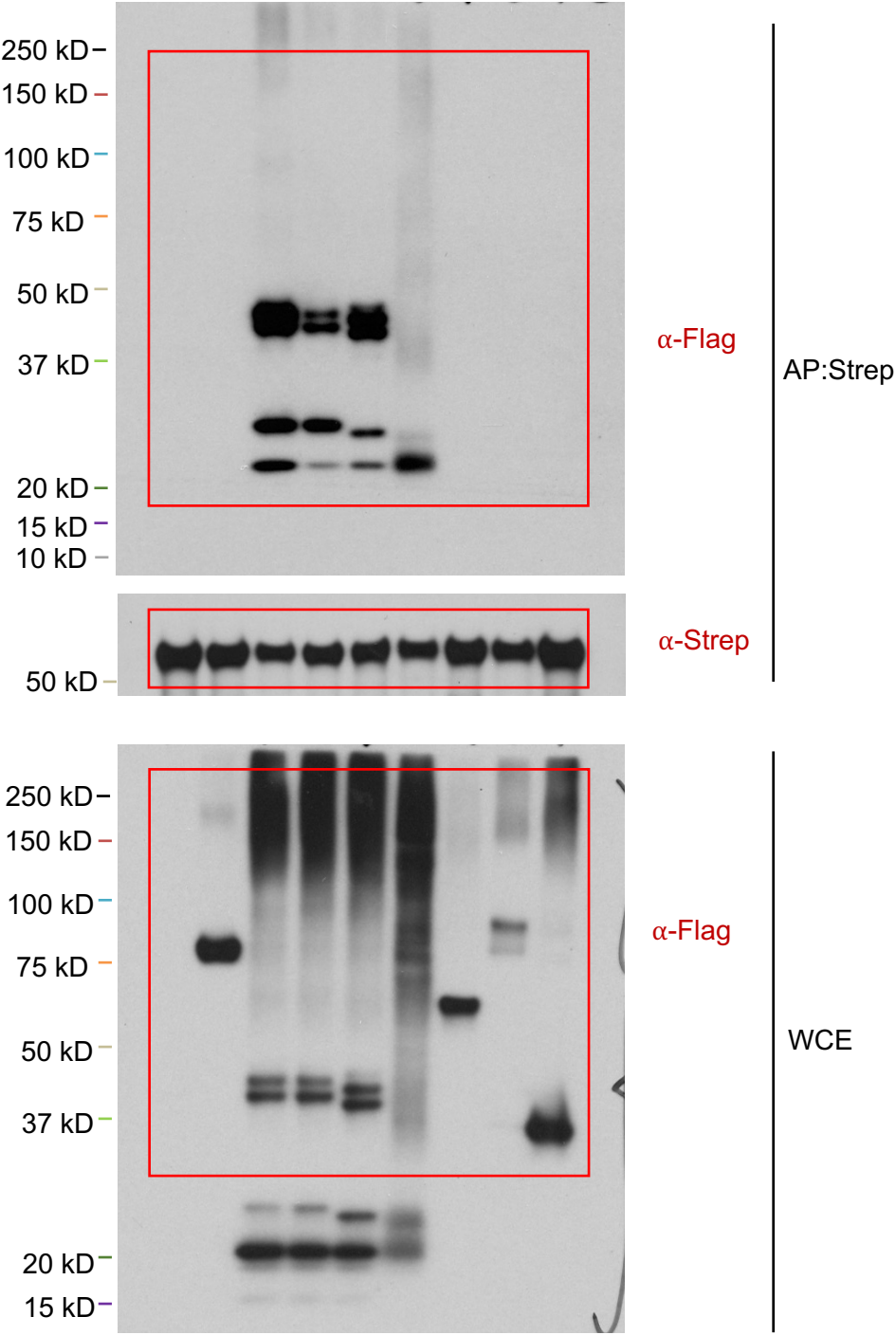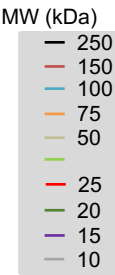

Supplement: Uncropped WB pictures — for both main figures and supplemental figures. [file jvi.00507-23-s0003.pdf]
