# Supplementary material for: The REEP5/TRAM1 complex binds SARS-CoV-2 NSP3 and promotes virus replication
Source: J Virol. 2023 Sep 28;97(10):e00507-23. doi: 10.1128/jvi.00507-23 (PMC10617467; doi:10.1128/jvi.00507-23)
Supplement: Fig. S2 — Colocalization of host-binding proteins with NSP3C and NSP4 at ROs. [file jvi.00507-23-s0004.docx]

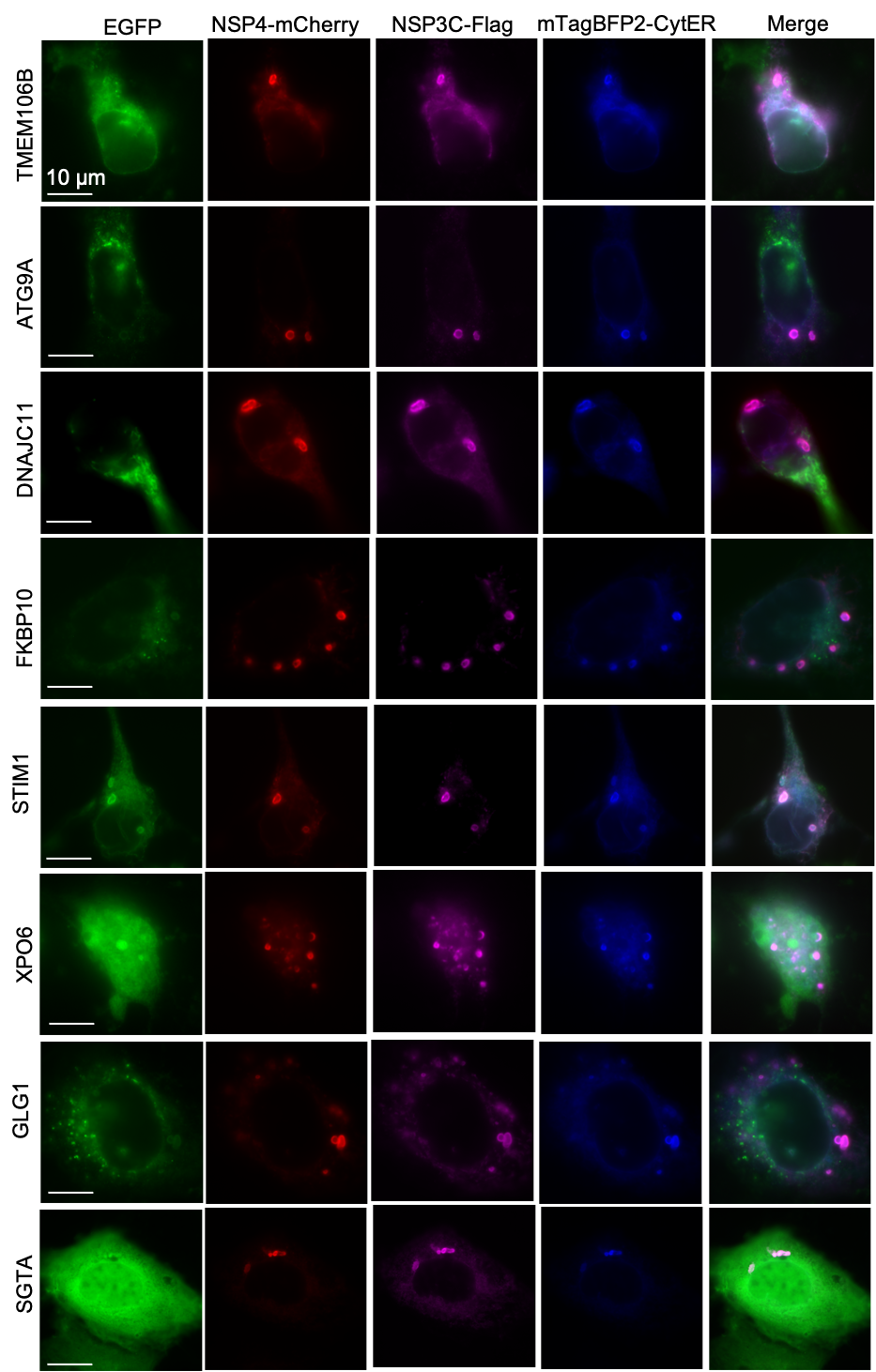


**Figure S2. Colocalization of host binding proteins with NSP3C and NSP4 at ROs.** Representative fluorescence images from U-2 OS cells expressing NSP3C-Flag, NSP4-mCherry and mTagBFP2-CytER were transfected with indicated plasmids tagged with EGFP. Scale bars: 10 μm.
