# Supplementary material for: The REEP5/TRAM1 complex binds SARS-CoV-2 NSP3 and promotes virus replication
Source: J Virol. 2023 Sep 28;97(10):e00507-23. doi: 10.1128/jvi.00507-23 (PMC10617467; doi:10.1128/jvi.00507-23)
Supplement: Fig. S3 — Validation of REEP5 and TRAM1 KO U-2 OS cells. [file jvi.00507-23-s0005.docx]

**
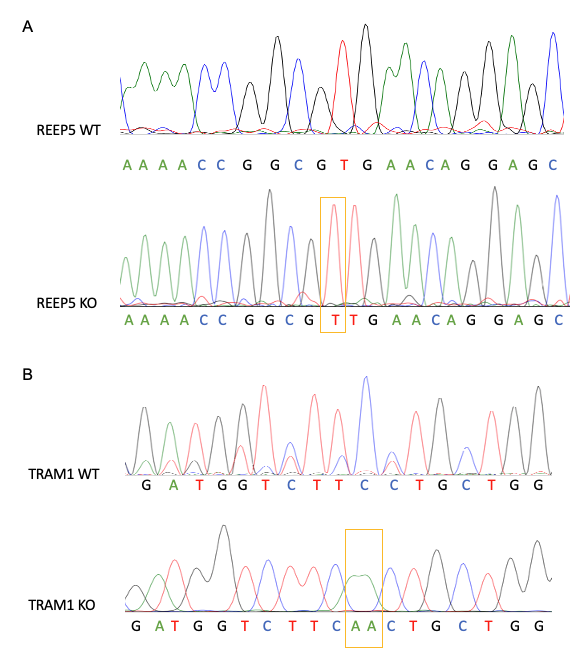
**

**Figure** **S3. Validation of REEP5 and TRAM1 KO U-2 OS cells.** Genomic DNA from REEP5 and TRAM1 KO U-2 OS cells were extracted and amplified by PCR. The sequencing results of PCR products from REEP5 KO (**A**) and TRAM1 KO (**B**) U-2 OS cells showed the frameshift mutation marked with yellow rectangle.
