# Supplementary material for: The REEP5/TRAM1 complex binds SARS-CoV-2 NSP3 and promotes virus replication
Source: J Virol. 2023 Sep 28;97(10):e00507-23. doi: 10.1128/jvi.00507-23 (PMC10617467; doi:10.1128/jvi.00507-23)
Supplement: Supplemental material legend — Checklist of supplemental material [file jvi.00507-23-s0006.docx]

**SUPPLEMENTAL MATERIAL**

SUPPLEMENTAL FIGURE S1-S3.

SUPPLEMENTAL TABLE S1.

Raw data table (provided as Excel files)

Key Reagents Table (provided as Excel files)

Uncropped immunoblot membranes (Main and supplementary figures)
